# Ultra‐Stable Topological Telluride Monolayers for Next‐Generation Battery Anodes and Sulfur Hosts

**DOI:** 10.1002/advs.202515841

**Published:** 2025-11-03

**Authors:** Shehzad Ahmed, Awais Ghani, Rashid Mehmood, Ahsan Ali, Naveed Hussain, Abdul Khaliq, Jun Han, Kemeng Ji, Danish Khan, Imran Muhammad

**Affiliations:** ^1^ China‐UK Low Carbon College Shanghai Jiao Tong University Shanghai 201306 P. R. China; ^2^ Smart Materials for Architecture Research Lab Innovation Center of Yangtze River Delta Zhejiang University Jiaxing 314100 P. R. China; ^3^ Department of Engineering Materials Engineering School of São Carlos University of São Paulo Avenue João Dagnone, 1100 São Carlos 13563‐120 Brazil; ^4^ Department of Physics Guangdong Technion Israel Institute of Technology Shantou Guangdong 515063 P. R. China; ^5^ Department of Electrical Engineering and Computer Science University of California Irvine Irvine CA 92697 USA; ^6^ Key Laboratory for Green Chemical Technology of Ministry of Education Collaborative Innovation Center of Chemical Science and Engineering (Tianjin) School of Chemical Engineering and Technology Tianjin University Tianjin 300350 P. R. China; ^7^ National Industry‐Education Platform of Energy Storage Tianjin University 135 Yaguan Road Tianjin 300350 P. R. China; ^8^ College of New Materials and New Energies Shenzhen Technology University Shenzhen Guangdong 518118 P. R. China

**Keywords:** high‐capacity anodes, molecular‐dynamics simulations, polysulfide anchoring, ternary telluride monolayers, topological materials

## Abstract

Rechargeable batteries are approaching the energy density ceiling set by conventional intercalation electrodes, while still suffering from the polysulfide shuttle and dendrite growth. Here, 2D ternary metal tellurides (HfTiTe_4_, ZrTiTe_4_, and HfZrTe_4_) are computationally designed, exhibiting a unique electronic environment with topological band structures and serving as multifunctional materials for ultrafast ion transport and strong catalytic anchoring in battery applications. Adsorption strengths demonstrate robust Li^+^/Na^+^ ion binding with considerable charge transfer, ensuring persistent chemisorption without affecting conductivity. Low ion‐diffusion barriers of 0.206 eV for Li^+^ and 0.046 eV for Na^+^, and ultrahigh theoretical capacities up to 1600 mAh g^‒1^ for Li^+^ and 1350 mAh g^‒1^ for Na^+^, high open‐circuit voltages in the range of 0.47–0.54 V for Li^+^ and 0.34–0.42 V for Na^+^ nominate them high‐energy anode materials. Additionally, these monolayers mitigate the shuttle effect by exhibiting high reactivity and charge redistribution for polysulfide anchoring. Thermodynamic and kinetic calculations for the sulfur reduction process show that HfZrTe_4_ possesses the lowest overpotential and activation barriers, while ZrTiTe_4_ and HfTiTe_4_ exhibit balanced binding and redox stability. This research on topological tellurides not only suggests them for next‐generation anodic applications but also for appealing anchoring materials for lithium‒sulfur cathodes.

## Graphical abstract

This study computationally designs 2D ternary metal tellurides (HfTiTe_4_, ZrTiTe_4_, and HfZrTe_4_) with unique electronic environments for battery applications. These materials offer low ion diffusion barriers, ultrahigh theoretical capacities, and robust ion binding, making them promising anodes. They also mitigate the polysulfide shuttle effect and show balanced binding and redox stability, suggesting their potential for next‐generation anodes and lithium‒sulfur cathodes.

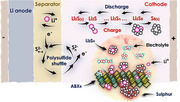

## 1 Introduction

Rechargeable lithium‐ion batteries (LIBs) revolutionized energy storage, becoming the foundation of portable electronics, electric vehicles, and grid‐scale systems.^[^ ^1^, ^2^ ^]^ Despite decades of progress, LIBs still face critical challenges in energy and power densities, escalating materials costs, and long‐term sustainability.^[^ ^3^ ^]^ The heavy reliance on expensive transition metals such as Ni and Co in conventional cathodes increases costs and threatens resource sustainability.^[^ ^4^, ^5^ ^]^ Consequently, the global academic and industrial communities are actively pursuing complementary or alternative energy‐storage technologies.^[^ ^6^ ^]^ Sodium–ion batteries (SIBs) have gained acceptance as an alternative due to their abundance, low cost, and chemical safety.^[^ ^7^ ^]^ Similar to LIBs, the electrochemical performance of SIBs is heavily reliant on the development of new electrode materials with high capacity, quick kinetics, and structural stability.^[^ ^8^ ^]^ Beyond intercalation chemistry,^[^ ^9^ ^]^ sulfur‐based batteries,^[^ ^10^ ^]^ including Li–S, Na–S, K–S,^[^ ^11^ ^]^ and multivalent metal–sulfur systems,^[^ ^12^ ^]^ have attracted intense attention owing to their theoretical energy density of up to 2 600 Wh kg^−1^.^[^ ^13^ ^]^ However, sulfur cathodes still suffer from three critical bottlenecks: i) low electronic and ionic conductivity; ii) ≈80% volumetric expansion during cycling, leading to structural pulverization; and iii) dissolution of intermediate polysulfides in ether‐based electrolytes, triggering the “shuttle effect” leading to anode corrosion and active sulfur loss.^[^ ^14^, ^15^ ^]^ To overcome these constraints, two‐dimentional (2D) materials have emerged as promising hosts and electrodes due to their ultrathin morphology, tunable surface chemistry, high conductivity, and mechanical flexibility.^[^ ^9^, ^16^ ^]^ Examples include graphene,^[^ ^9^, ^17^ ^]^ black phosphorene,^[^ ^18^ ^]^ borophene,^[^ ^19^ ^]^ and MXenes exhibit significant potential.^[^ ^20^ ^]^ Similarly, polar compounds such as metal oxides,^[^ ^21^, ^22^ ^]^ sulfides,^[^ ^23^ ^]^ carbides,^[^ ^24^ ^]^ and nitrides^[^ ^24^ ^]^ have been investigated as functional coatings or sulfur anchors to impede polysulfide migration.

Metal tellurides are promising next‐generation energy‐storage materials due to their high electrical conductivity, structural stability, and electronic tunability.^[^ ^25^ ^]^ Transition metal telluride, like MoTe_2_,^[^ ^26^ ^]^ NiTe_2_,^[^ ^27^ ^]^ CoTe_2_,^[^ ^28^ ^]^ and Bi_2_Te_3_, is a semimetallic or metallic material with higher electron mobility than chalcogenides. Tellurium has a higher electrical conductivity (2 × 10^2^ S m^‒1^) and adapts to volume changes,^[^ ^29^ ^]^ resulting in better mechanical stability and longer battery life than its sulfur‐ or selenium‐based counterparts.^[^ ^30^ ^]^ Beyond conventional tellurides, a distinct class of topological tellurides has attracted increasing interest due to their combined quantum and electrochemical functionality.^[^ ^31^ ^]^ Compounds such as Zr_2_Te_2_P,^[^ ^31^ ^]^ TlBiTe_2_,^[^ ^31^ ^]^ TaIrTe_4_,^[^ ^32^ ^]^ Bi_2_Se_2_Te,^[^ ^33^ ^]^ PbBi_2_Te_4_,^[^ ^34^ ^]^ TaPdTe_5_,^[^ ^30^ ^]^ NbFeTe_2_,^[^ ^35^ ^]^ and Ta_4_Pd_3_Te_16_^[^ ^36^ ^]^ with nontrivial band topology and robust Dirac or Weyl surface states. These compounds are highly conductive and structurally anisotropic, making them well‐suited for trapping polysulfides and facilitating ion diffusion. In Li–S and Na–S batteries, their layered structures, strong spin‐orbit interactions, and polarity make them versatile options for anodes and sulfur hosts.^[^ ^37^ ^]^ These features create opportunities for novel design strategies in the intersection of quantum materials and electrochemical energy storage. A newly identified class of topological ternary tellurides (TTMCs) with the formula ABX_4_ (A/B = Zr, Hf, and Ti; X = S, Se, and Te) combines topological surface states with layered structures and metallic conductivity.^[^ ^37^, ^38^ ^]^ These attributes establish a novel frontier for the development of high‐performance energy‐storage materials.

This study examines the three most stable transition‐metal chalcogenides, namely HfTiTe_4_, ZrTiTe_4_, and HfZrTe_4_. It carefully explores their potential as dual‐function energy‐storage materials: i) anodes for LIBs and SIBs and ii) effective S hosts in Li–S and Na–S batteries. Our thorough investigation assesses their stability, adsorption characteristics, electrical behavior, and redox compatibility, emphasizing their potential to address existing limitations in next‐generation energy‐storage devices.

## 2 Computational Details

All first‐principles calculations were performed with the Vienna ab initio Simulation Package (VASP 5.5.4),^[^ ^39^ ^]^ employing the projector‐augmented‐wave (PAW) method to describe electron–ion interactions.^[^ ^40^ ^]^ Exchange‐correlation effects were treated within the generalized gradient approximation (GGA) using the Perdew‐Burke‐Ernzerhof (PBE) functional.^[^ ^40^, ^41^ ^]^ Long‐range van der Waals (vdW) interactions were included via the Grimme D3 dispersion correction scheme.^[^ ^42^ ^]^ A plane‐wave energy cutoff of 550 eV was adopted for all systems.^[^ ^43^ ^]^ Brillouin‐zone sampling was carried out on Monkhorst–Pack k‐point meshes,^[^ ^43^ ^]^ employing a 14 × 18 × 1 grid for the primitive cell and maintaining an equivalent k‐point density for supercell calculations to ensure convergence. The energy convergence criterion for electronic self‐consistency was converged to within 1 × 10^−5^ eV, and the residual Hellmann–Feynman force on each atom was restricted to ≤ 0.01 eV Å^−1^ during structural relaxation.^[^ ^43^ ^]^

Both spin‐orbit coupling (SOC) and non‐SOC configurations were evaluated for comparison. To capture relativistic effects in band‐structure calculations, SOC and noncollinear magnetism were included.^[^ ^44^ ^]^ Chemical bonding characteristics and orbital‐resolved electronic interactions were analyzed using the crystal orbital Hamilton population (COHP) method in the LOBSTER code.^[^ ^45^, ^46^ ^]^ The climbing‐image determined minimum‐energy pathways and ion‐migration barriers for Li^+^/Na^+^ diffusion nudged elastic band (CI‐NEB) technique; each path was discretized into at least five intermediate images until the maximum force component fell below 0.05 eV Å^−1[^ ^47^ ^]^. Thermal stability was assessed by ab initio molecular dynamics (AIMD) simulations in the canonical (NVT) ensemble with a Nosé‐Hoover thermostat.^[^ ^48^ ^]^ Simulations were conducted at 300 and 500 K with a time step of 1 fs, recording energies and atomic trajectories every 10 fs. We monitored potential‐energy fluctuations and structural integrity to confirm thermal robustness (see Figures S1–S4, Supporting Information for complete trajectories).

## 3 Results and Discussion

### 3.1 Structural Characteristics

**Figure 1:**
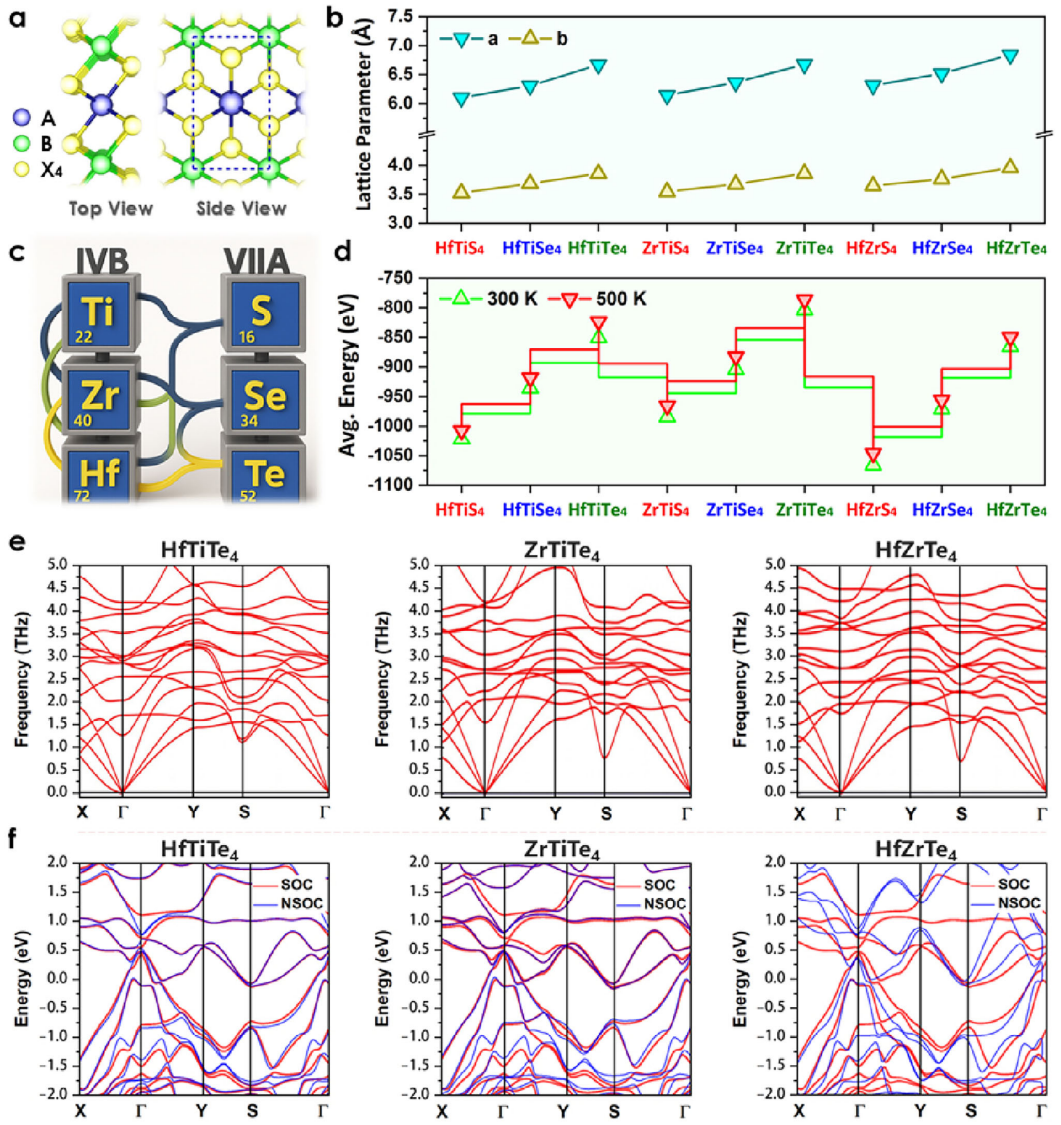
Structural and electronic properties of 2D topological TTMCs. a) Top and side views of nine ABX_4_ (A/B = Ti, Zr, or Hf; X = S, Se, or Te) monolayers derived from 1T‐MX_2_. b) Lattice constants versus chalcogen covalent radius showing Vegard‐like trends. c) Combinatorial design tree of ABX_4_ stoichiometries. d) AIMD of average potential energies at 300  and 500 K showing Te‐phase thermal stability. e) Phonon spectra confirming dynamic stability. f) Topological band structures of HfTiTe_4_, ZrTiTe_4_, and HfZrTe_4_ calculated using GGA with SOC (red lines) and without SOC (blue lines).

Monolayer TTMCs obey the stoichiometric formula ABX_4_, where A/B = Ti, Zr, or Hf and X = S, Se, or Te. The compositional matrix yields nine distinct phases.^[^ ^38^ ^]^ All monolayers were generated by doubling the 1 × 2 1T‐MX_2_ primitive cell along the *a*‐axis. One transition‐metal site with a second 4*d*/5*d* element (**Figure** **1**a). This construction preserves the overall ABX_4_ symmetry while introducing intrinsic lattice asymmetry that is key to the topological band inversion reported earlier. Optimized in‐plane lattice parameters (Figure 1b) vary linearly with the chalcogen covalent radius: *a* = 6.65–6.92 Å for sulfides, 7.02–7.31 Å for selenides, and 7.58–7.89 Å for tellurides, consistent with Vegard‐like behavior across the IVB metal series.^[^ ^49^ ^]^ The combinatorial construction scheme is illustrated in Figure 1c. To substantiate thermal stability, AIMD simulations were carried out at 300 and 500 K for 10 ps under the NVT ensemble (Figure 1d; Figures S1‒S4, Supporting Information). We use phonon dispersion spectra to check the dynamic stability of HfTiTe_4_, ZrTiTe_4_, and HfZrTe_4_ monolayers (Figure 1e). None of the three structures exhibits imaginary phonon modes within the Brillouin zone, signifying exceptional dynamic stability and lattice integrity. This confirms that monolayers are stable, making them promising for electronic and energy‐storage applications. Furthermore, the electronic band structures of HfTiTe_4_, ZrTiTe_4_, and HfZrTe_4_ confirm their topological metallicity (Figure 1f). SOC introduces pronounced band splitting near the Fermi level, especially at the Γ and S points. HfTiTe_4_ exhibits the strongest SOC‐induced reconstruction, highlighting the potential for Dirac surface states. ZrTiTe_4_ retains semi‐metallic behavior even after SOC is included; we found moderate SOC effects compared to HfZrTe_4_. The dynamic stability of ABTe_4_‐type telluride monolayers makes them a strong candidate for future quantum, thermoelectric, and alkali‐ion battery applications.^[^ ^50^ ^]^ Consequently, the conductive HfTiTe_4_, ZrTiTe_4_, and HfZrTe_4_ are selected as the model systems for detailed electrochemical investigation. In addition to their thermal and dynamical stabilities, the monolayer ternary transition metal tellurides (HfTiTe_4_, HfZrTe_4_, and ZrTiTe_4_) show a finite density of states (DOS) at the Fermi level in all three cases.

**Figure 2:**
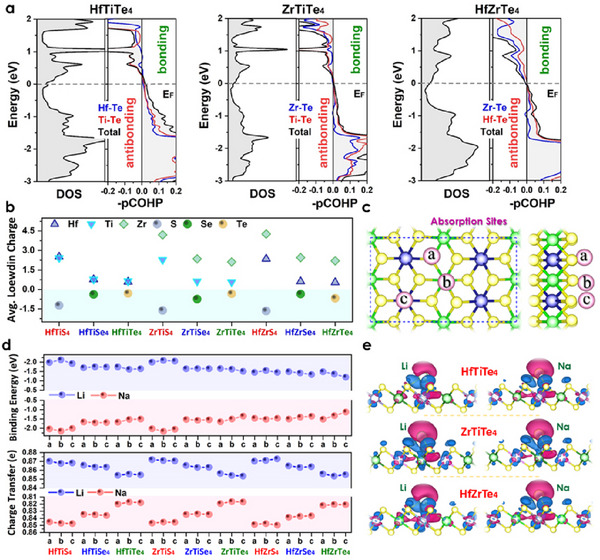
Chemical stability, ion transport, and alkali adsorption behavior of monolayer TTMCs. a) Total DOS, COHP, and projected COHP of HfTiTe_4_, ZrTiTe_4_, and HfZrTe_4._ The analyses show robust metallicity and finite DOS at *E*_n_ even after Li^+^/Na^+^ ions adsorption. b) Average Löwdin charges for each atom in the monolayer ABX_4_ structure. The charge distribution reflects the degree of charge among constituent atoms. c) Top and side views of a 2 × 2 × 1 supercell with three high‐symmetry adsorption sites. d) Site‐dependent binding energies and Bader charge transfer per site of Li^+^/Na^+^ ions on ABX_4_ surfaces. e) Charge‐density‐difference isosurfaces (± 0.002 e Å^‒3^). These images visualize electron accumulation (blue) and depletion (red) upon Li^+^/Na^+^ adsorption on tellurides.

Contributions near the Fermi energy mainly arise from the *d*‐orbitals of transition metals and the *p*‐orbitals of Te, indicating strong orbital hybridization (Figure S5, Supporting Information). The addition of spin‐orbit coupling (SOC) alters the electronic distribution, strengthening the states near E*_F_* and confirming that these monolayers have a topological structure. Their chemical stability is further supported by COHP bonding curves (**Figure** **2**a), which reveal a well‐ordered atomic arrangement. Furthermore, these monolayers show more favorable bonding compared to their sulfide and selenide counterparts, as indicated by the geometry and average Löwdin charge (Figure 2b). Our calculations confirm that the chemical stabilities of HfTiTe_4_, HfZrTe_4_, and ZrTiTe_4_ structures are well established by phonon bands and their dynamical stabilities together make them promising candidates for fast charge transport as electrode materials.^[^ ^51^ ^]^

### 3.2 Li^+^/Na^+^ Ions Adsorption Energetics

To evaluate the suitability of ABX_4_ monolayers as high‐rate anodes. A single Li^+^/ Na^+^‐ion was placed on three symmetrically distinct sites (Figure 2c) in 3 × 3 × 1 supercells of HfTiTe_4_, ZrTiTe_4_, and HfZrTe_4_: a) atop the B‐metal site, b) atop the chalcogen X, and c) atop the A‐metal site. The adsorption energy (*E_ad_*) is defined as:

(1) $$E_{ad}=E_{M+\mathrm{AB}X_{4}}-E_{\mathrm{AB}X_{4}}-E_{M}$$

where *E*_M+ABX4_ is the total energy of the monolayer ABX_4_ with an adsorbed atom M, *E*_ABX4_ is the total energy of the pristine ABX_4_ monolayer, and *E*_4_ is the cohesive energy per Li^+^/ Na^+^‐ion in its bulk phase. All nine monolayers exhibit negative *E*_ad_ (Figure 2d), confirming that spontaneous adsorption is a key requirement for stable ion retention during battery cycling. The *E_ad_* quantifies the thermodynamic favorability of ion adsorption. In our results, tellurides such as HfTiTe_4_, ZrTiTe_4_, and HfZrTe_4_ exhibit comparatively high Li and Na adsorption energies (|*E_ad_* | ≈1.2–1.7 eV). These high values indicate robust ion–surface interactions, ensuring stable adsorption during cycling and preventing ion clustering or detachment. Among the studied tellurides, ZrTiTe_4_ shows the strongest Li^+^ binding (≈1.66 eV), while HfTiTe_4_ shows energetically stable *E_ad_* values that enable both stability and rapid diffusion. Site‐projected analysis reveals that the site‐b is the most favorable across all cases, offering threefold Te coordination and maximum overlap with the metal *d*_z2_ orbital. This energetics guarantees a uniform ion distribution and effectively inhibits dendrite nucleation or metallic clustering even at elevated loadings.

Projected density of states (PDOS, Figures S6 and S7, Supporting Information) reveal that the monolayers retain their metallic character after Li^+^/Na^+^ ions uptake. In the low‐coverage limit, the Li‐2*s* and Na‐3*s* states are weakly split and hybridize with the Te‐5*p* and metal‐*d* bands within ± 0.5 eV of the Fermi level. At full coverage (*x* ≈3 in M*_x_* ABX_4_), the alkali *s*‐states broaden and partially fill the conduction region, increasing the total DOS at *E*_F_ by ≈30%. This continuous metallic pathway ensures low interfacial resistance and facile electron transport during rapid (de‐)lithiation or (de‐)sodiation. To further understand stability, we performed Bader charge and pCOHP analyses (Figure 2d). A substantial charge transfer from Li^+^/Na^+^ ions to the telluride surface, with typical values ranging from 0.81 to 0.87 |e| for Li^+^ and 0.82 to 0.85 |e| for Na^+^. This significant charge donation arises from the substantial electronegativity disparity between alkali metals and tellurium, along with the polar characteristics of the metal‐tellurium bonds. The accompanying charge‐density difference (Δρ) maps ($\Delta \rho =\rho_{\mathrm{MAB}X_{4}}\;-\rho_{M\;}-\rho_{\mathrm{AB}X_{4}}$ Figure 2e) display pronounced electron depletion around the alkali ion and concurrent accumulation within the interfacial Te *p*‐orbitals, forming a polarized “ionic cushion” that screens Coulomb repulsion between adjacent *ad‐*ions.

Different bonding reconfiguration mechanisms controlling the adsorption stability of Li^+^/Na^+^ ions in ABX_4_ monolayers are revealed by pCOHP analysis (Figure S8, Supporting Information). The exceptional stability of topological tellurides (HfTiTe_4_, ZrTiTe_4_, and HfZrTe_4_) upon Li^+^/Na^+^ intercalation is demonstrated by i) the absence of antibonding states near *E*_F_ (± 0.3 eV) and ii) the preservation of metal–Te covalency. On the other hand, repulsive A/B‐X interactions (Hf–S, Zr–S, and Ti–S; Hf–Se, Zr–Se, and Ti–Se bonds at *E*_F_) cause sulfides/selenides to undergo electronic destabilization through Fermi‐level penetration into antibonding manifolds (Δ*E*_F_ = −0.15 to −0.32 eV). Interestingly, HfTiSe_4_ exhibits intermediate stability, retaining its bonding properties after adsorbing Li^+^/Na^+^ ions. A bond‐resolved investigation found that tellurides contain strong metal–Te bonding without antibonding states around *E*_F_, while sulfides and selenides destabilize because of antibonding interactions in significant metal–chalcogen bonds.^[^ ^52^ ^]^ The stable bonding of telluride monolayers makes them promising anodes for Li^+^/Na^+^ batteries. This shows how vital chalcogen selection is for structural integrity during electrochemical cycling. This interaction between robust chemisorption and retained metallic conductivity underpins the exceptional rate capability predicted for these telluride anodes.^[^ ^53^ ^]^

### 3.3 Anchoring of Li^+^/Na^+^ Polysulfides

**Figure 3:**
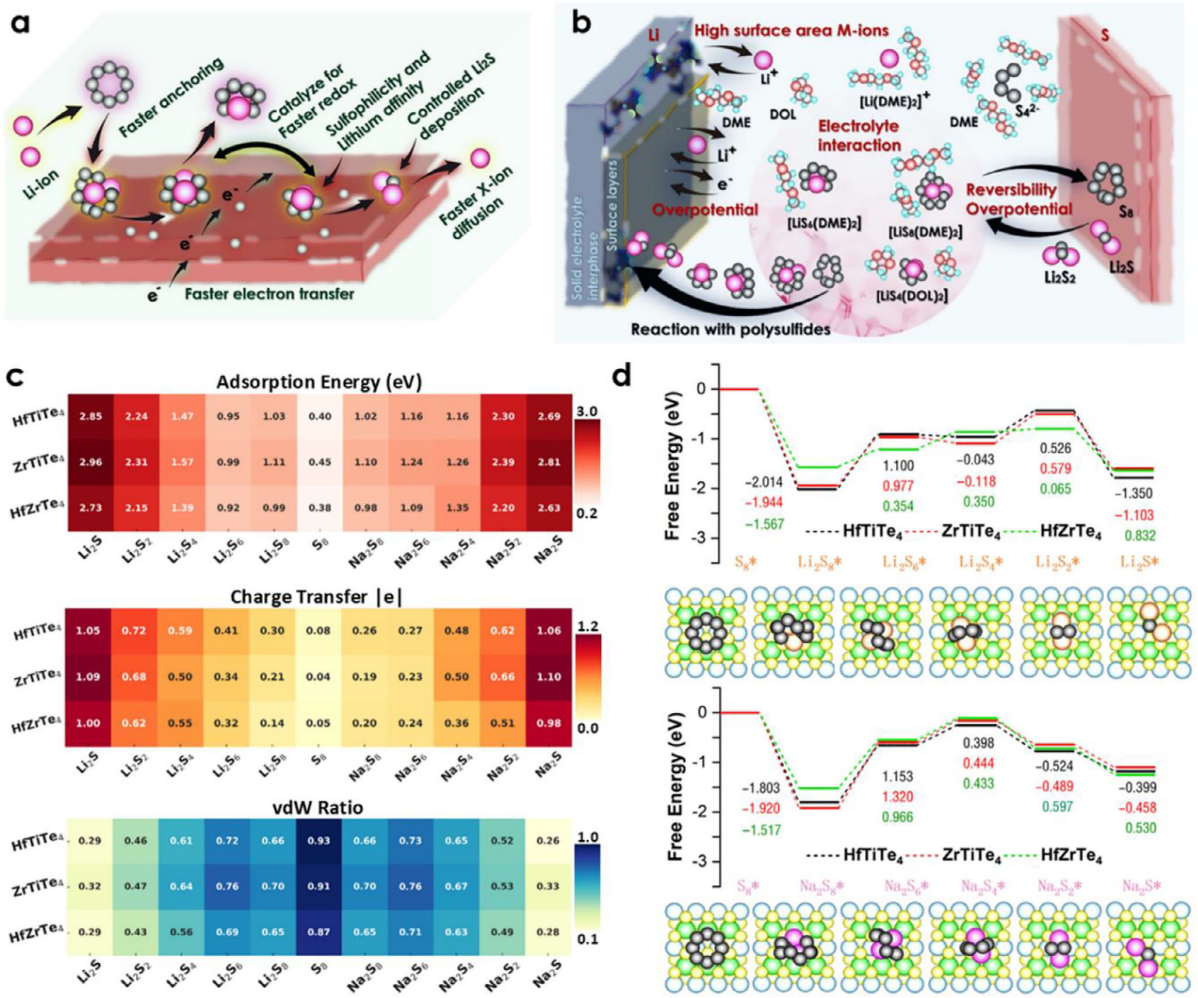
Sulfur‐cathode chemistry and anchoring efficacy of ABTe_4_. a) Schematic of the Li–S cell illustrating electron/ion flow during discharge. b) Redox cascade: S_8_ → Li_2_S_8_ → Li_2_S_6_ → Li_2_S_4_ → Li_2_S_2_ → Li_2_S. Dissolution of long‐chain polysulfides in DOL/DME causes the shuttle effect. ABTe_4_ offers competitive advantages including viable ion mobility, redox catalysis, controlled polysulfide deposition, and strong anchoring of long‐chain polysulfide clusters. c) Heatmaps of TTMC anchoring mechanisms. Top: Short‐chain Na_2_S_n_/Li_2_S_n_ exhibits strongest adsorption on HfZrTe_4_. Mid: Charge transfer decreases from Li_2_S/Na_2_S (≈1 |e|) to S_8_ (≈0.05 |e|), showing chemisorption‐physisorption transition. Bottom: *vdW* dominates for long chains (≈80%), while chemisorption prevails for Li_2_S (≈20%). HfZrTe_4_ optimally balances chemical bonding (to suppress shuttling) and physical adsorption (to accommodate S_8_), making it ideal for Li–S cathodes. d) Stepwise Gibbs free‐energy profiles for S_8_ → Li_2_S reduction on each monolayer. HfZrTe_4_ exhibits the lowest over‐potential (0.934 eV), highlighting its superior catalytic performance.

The working mechanism of the sulfur electrode is schematically shown in **Figure** **3**a. The multi‐electron transfer reaction 16M + S_8_ → 8M_2_S in the sulfur cathode provides numerous advantages over traditional alkali metal‐ion batteries. Figure 3b illustrates the reaction process of Li^+^ with polysulfides leading to the formation of metallic polysulfides over cathode materials under the influence of liquid electrolytes, particularly dimethoxyethane (DME) and 1,3‐dioxolane (DOL).^[^ ^29^ ^]^ These solvents can interact with M_2_S_n_, mitigating side reactions and suppressing the formation of gaseous byproducts such as H_2_S. We designed the TTMC monolayer to effectively suppress the shuttle effect by constraining the dissolution of Li_2_S_n_. When paired with anchoring materials, these strong interactions result in efficient charge‐discharge and high capacity in Li–S batteries. Next, we explored the anchoring capabilities of sulfur‐based materials (HfTiTe_4_, ZrTiTe_4_, and HfZrTe_4_). We adsorbed the S_8_ cluster and Li_2_S_n_/Na_2_S_n_ species (*n* = 1, 2, 4, 6, and 8) on these materials, showing a descending trend in adsorption energies *E*_ad_ (Figure 3c). Importantly, S_8_ and M_2_S_n_ exhibit energetically stable, exothermic interactions with the monolayer (see schematic adsorption illustration in Figure S9, Supporting Information). Comparison with liquid electrolytes show that long‐chain Li_2_S_n_/Na_2_S_n_ bind more strongly to our anchoring materials, indicating effective shuttle suppression (Figure 3c). Electrolytes like DME and DOL exhibit relatively weak binding (0.77–0.85 or 0.60–0.90 eV) which is insufficient to suppress the shuttle effect. Carbon‐based hosts like graphene, C_3_B, C_3_N, and C_5_N improves adsorption strength, C_5_N binds Li_2_S_8_ most strongly (≈1.25 eV).^[^ ^54^ ^]^ Transition metal dichalcogenides (TMDs) like TiS_2_ and VS_2_ outperform carbon materials, with Li_2_S_4_ reaching 2.42 eV and Na_2_S_4_ reaching ≈2.20 eV in VS_2_.^[^ ^55^, ^56^ ^]^ Boron–graphdiyne (BGDY) offers moderate anchoring.^[^ ^57^ ^]^ Na‐polysulfides, particularly Na_2_S_4_ (≈3.01 eV), bond strongly to MoS_2_. In contrast, Fe@rg‐C_3_N_4_ bonds well to Na_2_S_4_ (≈2.70 eV) and Na_2_S_8_ (≈2.50 eV). Heteroatom‐doped carbons and TMDs, such as VS_2_, MoS_2_, and Fe@rg‐C_3_N_4_, offer superior binding energies for polysulfides compared to electrolytes and pure carbon materials.^[^ ^58^ ^]^ To prevent polysulfide dissolution, reduce the shuttle effect, and stabilize Li–S and Na–S batteries, stronger chemisorption is needed. The performance of Li–S and Na–S clusters as sulfur hosts depends on their interaction with HfTiTe_4_, ZrTiTe_4_, and HfZrTe_4_ monolayers, which exhibit adsorption strengths, charge redistribution patterns, and *vdW* contributions. ZrTiTe_4_ consistently outperforms the other two monolayers, with adsorption energies of 2.96 eV for Li_2_S and 2.81 eV for Na_2_S, as shown in Figure 3c. HfZrTe_4_ records somewhat lower adsorption energies but maintains values adequate for efficient anchoring; however, HfTiTe_4_ exhibits slightly lower adsorption energies, determining the strong binding with outstanding electrochemical stability.

Charge‐transfer analysis shows that HfTiTe_4_ enhances electrostatic stability and maintains a mostly ionic interface by promoting the highest electron donation from adsorbed clusters. This corresponds to 0.72 |e| for Li_2_S and 0.68 |e| for Na_2_S. Moreover, ZrTiTe_4_ and HfZrTe_4_ show significant charge transfer, maintaining conductivity while promoting robust ionic anchoring. We also evaluated the medium‐chain case by calculating the charge‐density difference (*Δρ*) for Li_2_S_4_ and Na_2_S_4_ clusters adsorbed on these monolayers (Figure S10, Supporting Information). The results show that anchoring materials significantly gain charge from the middle‐chained Li_2_S_4_/Na_2_S_4_ polysulfides directing the electrochemical capability of host materials.

The electronic structure investigations (Figures S11–S16, Supporting Information) demonstrate pronounced charge redistribution and orbital hybridization between S 3*p* and metal‐*d*/*p* states, engendering new electronic states proximal to the Fermi level. The topological Dirac‐like surface states form an extended π‐network that delocalizes interfacial charge, thereby lowering the nucleation barrier for Li_2_S/Na_2_S and accelerating redox kinetics. The contribution of S near the Fermi level arises from occupied states of S lone pairs and S‐metal bonds, as well as from unoccupied states of antibonding orbitals, thereby enhancing electronic conductivity and facilitating polysulfide immobilization. The *vdW* ratios indicate the balance between chemical and physical adsorption. For intermediate polysulfides like Li_2_S_6_ and Li_2_S_4_, HfTiTe_4_ and ZrTiTe_4_ exhibit larger *vdW* contributions, suggesting a mixed binding mechanism that permits structural flexibility while maintaining stability. HfZrTe_4_ binds polysulfides more strongly but offers less structural flexibility during redox conversion due to lower *vdW* ratios, indicating chemisorption‐dominated interactions.

All three topological tellurides are strong candidates for high‐efficiency Li–S and Na–S batteries, as they possess distinct properties that complement one another. ZrTiTe_4_ has the strongest binding, HfTiTe_4_ has the best charge transfer, and HfZrTe_4_ has a surface that facilitates faster ion diffusion. Figure 3d schematically illustrate the ideal characteristics of the anchoring material, including the effective redox reaction from S_8_ to M_2_S catalyzed by HfTiTe_4_, ZrTiTe_4_, and HfZrTe_4_ substrates, thereby strengthening the anchoring of metal polysulfides and facilitating faster polysulfide diffusion across the electrode surface. The dominance of physical interaction can be seen for long‐chain Li_2_S_n_/Na_2_S_n_ (*n* = 6 and 8) and S_8_ clusters, and a more strengthened chemical interaction is observed in short‐chain Li_2_S_n_/Na_2_S_n_ (*n* = 1, 2, and 4) polysulfides. These interaction trends between short‐ and long‐chain polysulfides are consistent with previous studies.

Energy decomposition (Figure 3d) reveals that short Li_2_S_n_/Na_2_S_n_ (*n* = 1–4) engage primarily in chemisorption mediated by *σ*‐type S–metal hybridization, a conclusion supported by PDOS analysis indicating significant orbital overlap between sulfur and metal atoms. In contrast, long‐chain species (*n* = 6 and 8) and S_8_ are predominantly stabilized by dispersive interactions, as evidenced by their weaker adsorption energies. These *vdW* contributions are sufficient to effectively immobilize polysulfides against concentration‐driven diffusion. The electrochemical conversion of sulfur on HfTiTe_4_, ZrTiTe_4_, and HfZrTe_4_ proceeds in multiple reduction steps, starting from elemental sulfur (S_8_) and ending with fully reduced metal sulfides (Li_2_S or Na_2_S). The sulfur‐reduction‐reaction (SSR) sequence was mapped via the elementary steps^[^ ^59^ ^]^:

(2) $$S_{8}^{*}+16M+2e^{-}\to M_{2}S_{8}^{*}+14\;M\;$$

(3) $$M_{2}S_{8}^{*}+14M+2e^{-}\to M_{2}S_{6}^{*}+M_{2}S_{2}+12\;M\;$$

(4) $$M_{2}S_{6}^{*}+M_{2}S_{2}+12M+2e^{-}\to M_{2}S_{4}^{*}+2M_{2}S_{2}+10\;M\;$$

(5) $$M_{2}S_{4}^{*}+2M_{2}S_{2}+10M+2e^{-}\to M_{2}S_{2}^{*}+3M_{2}S_{2}+8\;M\;$$

(6) $$M_{2}S_{2}^{*}+3M_{2}S_{2}+8M+8e^{-}\to M_{2}S^{*}+7M_{2}S$$

The corresponding Gibbs free‐energy (*ΔG*) profile (Figure 3d) shows that the overall S_8_ → Li_2_S transformation exhibits Δ*G* < 0.8 eV per electron, whereas the S_8_ → Na_2_S sequence is ≈0.3 eV less favorable. Among the three monolayers, HfZrTe_4_ exhibits the lowest overpotential (0.934 eV for Li_2_S_4_ → Li_2_S), indicating its superior catalytic activity. For Li–S systems, the first lithiation step (S_8_ → Li_2_S_8_) is strongly exergonic (*ΔG* = −1.57 to −2.01 eV), confirming that the process begins spontaneously. The second step (Li_2_S_8_ → Li_2_S_6_) is the most energetically demanding, particularly for HfTiTe_4_ and ZrTiTe_4_, where *ΔG* exceeds 0.95 eV, while HfZrTe_4_ shows a much lower penalty (≈0.35 eV), indicating an advantage in stabilizing long‐chain polysulfides. The next stage (Li_2_S_6_ → Li_2_S_4_) is nearly thermoneutral for HfTiTe_4_ and ZrTiTe_4_ but slightly uphill for HfZrTe_4_. Reduction from Li_2_S_4_ to Li_2_S_2_ again requires moderate energy input (0.33–0.59 eV), after which the final conversion to Li_2_S is strongly favorable (−0.83 to −1.35 eV), ensuring complete reduction once short‐chain species form. For Na–S systems, the overall pathway is similar but with generally smaller driving forces in the first step. S_8_ → Na_2_S_8_ remains spontaneous (*ΔG* = −1.52 to −1.92 eV) across all tellurides, but Na_2_S_8_ → Na_2_S_6_ shows higher energy requirements than the lithium counterpart, especially for ZrTiTe_4_ (+1.32 eV). The Na_2_S_6_ → Na_2_S_4_ transformation is moderately uphill (≈0.40–0.44 eV), followed by a downhill Na_2_S_4_ → Na_2_S_2_ step and a favorable final reduction to Na_2_S. Compared with other candidates, HfZrTe_4_ exhibits the smoothest free‐energy landscape, notably lowering barriers to high‐energy polysulfide steps, which are critical for suppressing shuttle and achieving uniform discharge. For comparison, we also evaluated the anchoring capabilities of 2D ternary metal sulfides and selenides. Motivated by the potential reduction of lithium and sodium polysulfides on HfZrTe_4_, we selected HfZrS_4_ and HfZrSe_4_ monolayers. These substrates displayed stable adsorption energies for S_8_ and various Li_2_S_n_/Na_2_S_n_ (n = 1, 2, 4, 6, and 8) species, effectively curbing the shuttling effect as shown in Figure S17 (Supporting Information). Moreover, we confirmed the electrochemical interaction between the substrates and medium‐chain polysulfides via Bader charge integration and charge‐density difference calculations, with results presented in Figure S18 (Supporting Information).

The Gibbs free‐energy profiles for HfZrS_4_ and HfZrSe_4_ show distinct stabilization trends for lithium and sodium polysulfides. HfZrS_4_ favors early‐stage lithium species like Li_2_S_2_ (−1.26 eV) and high‐sulfur sodium species such as Na_2_S_8_ (−1.64 eV). In contrast, HfZrSe_4_ stabilizes later‐stage intermediates like Li_2_S (−1.59 eV) and Na_2_S_2_ (−1.78 eV). HfZrS_4_ generally prefers early stages, except for a small energy barrier in the Li_2_S_2_ → Li_2_S transition (+0.23 eV). HfZrSe_4_ shows a slight uphill trend in the Li_2_S_8_ → Li_2_S_6_ transition (+0.22 eV) but strongly stabilizes the Li_2_S_4_ → Li_2_S_2_ transition (−0.59 eV). These results highlight complementary roles for HfZrS_4_ and HfZrSe_4_ in capturing different stages of polysulfide reduction (Figure S19, Supporting Information). Within tellurides, ZrTiTe_4_ provides a balanced pathway for both Li–S and Na–S, while HfTiTe_4_ strongly activates S_8_, promoting rapid reaction onset. We looked at the thermodynamic and kinetic characteristics of the M–S conversion routes on HfTiTe_4_, ZrTiTe_4_, and HfZrTe_4_ monolayers for polysulfide reduction. The thermodynamic overpotential (*η*) was characterized as the highest per‐electron positive Gibbs free‐energy variation in the incremental discharge sequence defined as:

(7) $$\eta \;=\;\max_{i}\left(\frac{\Delta G_{i}^{>0}}{n_{e,i}}\right)$$

where $\Delta G_{i}^{>0}$ is the free‐energy change for uphill steps, and *n*_*e*,*i*_ is the number of electrons transferred in step *i*. Since 1 eV per electron equals 1 V, this yields a direct voltage value.^[^ ^60^ ^]^ For Li–S systems, the *η* values are 0.55 V (HfTiTe_4_), 0.49 V (ZrTiTe_4_), and 0.18 V (HfZrTe_4_). The last one shows the most energetically beneficial conversion. The *η* values for Na–S are a little higher: 0.58, 0.66, and 0.48 V, but the trend stays the same, showing that HfZrTe_4_ is the most thermodynamically efficient. From a kinetic standpoint, the overall reaction pathway follows the canonical route: S_8_ → M_2_S_8_ → M_2_S_6_ → M_2_S_4_ → M_2_S_2_ → M_2_S (M = Li^+^/Na^+^). The Δ*G* value for each step was calculated using the equation:

(8) $$\Delta G=G_{\mathrm{product}}-G_{\mathrm{reactant}}$$

To estimate kinetic feasibility, activation energies (*E_a_*) were derived using the Brønsted‐Evans‐Polanyi (BEP) relation: *E_a_* = *α*Δ*G* + *β*, where *α* = 0.5 and *β* = 0.3 eV were employed to approximate surface‐mediated reaction barriers.^[^ ^61^ ^]^ Determined *E_a_* values designate the rate‐determining step (RDS) as M_2_S_8_ → M_2_S_6_ across all systems. For Li–S, *E_a_* goes down from 0.85 eV (HfTiTe_4_) to 0.477 eV (HfZrTe_4_). For Na–S, it goes down from 0.960 eV (ZrTiTe_4_) to 0.783 eV (HfZrTe_4_). These reductions mean that polysulfide conversion rates are orders of magnitude faster, notably for lithium. HfZrTe_4_ offers the best balance of performance, using the least amount of bias while allowing polysulfide to change quickly. This dual benefit reduces the buildup of intermediates, stops the shuttle effect, and encourages efficient cycling, making it a good sulfur host for both Li–S and Na–S batteries.

### 3.4 Li^+^/Na^+^ Ions and Li_2_S/Na_2_S Polysulfide Diffusion

**Figure 4:**
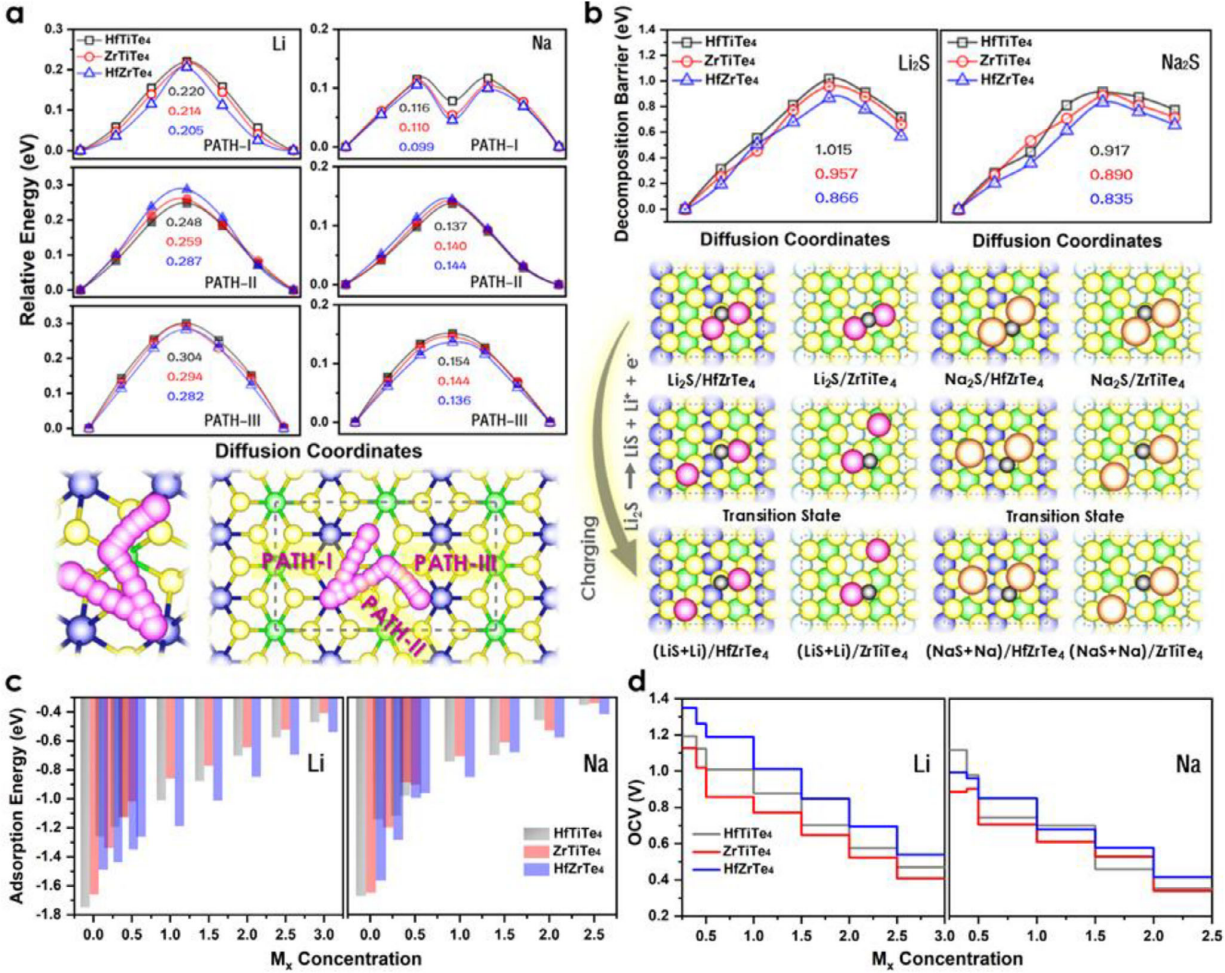
Li^+^/Na^+^ ion diffusion kinetics and cluster dissociation on TTMC monolayers. a) Composition‐dependent barrier comparison and corresponding schematics of the three migration pathways (I–III) for Li^+^/Na^+^ on HfTiTe_4_, ZrTiTe_4_, and HfZrTe_4_. Path I exhibits barriers of 0.205–0.220 eV for Li^+^ and 0.099–0.116 eV for Na^+^. b) Geometric snapshots and corresponding diffusion energies for Li_2_S and Na_2_S dissociation on the same surfaces, demonstrating energetically favorable surface‐mediated nucleation of the final discharge products. The concentration‐dependent electrochemical performance of TTMC monolayers is also shown. c) Declining adsorption energies as the number of Li^+^/Na^+^ ions increases (*x* = 0.0–3.0). Accordingly, achievable specific capacities vs Li^+^/Na^+^ ions concentration reach 1600, 1350, and 1200 mAh g^−1^ for HfTiTe_4_, ZrTiTe_4_, and HfZrTe_4_, respectively. d) Positive OCVs trends for Li^+^ and Na^+^ intercalation across the entire lithiation/sodiation range.

Ion mobility dictates rate performance. The diffusion energetics of Li^+^/Na^+^ ions through ternary telluride monolayers (HfTiTe_4_, ZrTiTe_4_, and HfZrTe_4_) along three different diffusion pathways (Paths I–III, **Figure** **4**a) were identified on each monolayer and quantified by CI‐NEB. Path‐I is the best way for lithium to move because HfZrTe_4_ has the lowest energy barrier (0.206 eV), followed by ZrTiTe_4_ (0.214 eV) and HfTiTe_4_ (0.220 eV). Path II has barriers that are 20%–30% higher than those of Path I. Path III has the most challenging route, with barriers exceeding 0.300 eV for HfTiTe_4_. This suggests a degree of diffusion anisotropy. Sodium ions are even better at moving around, as shown by the very low barrier of only 0.046 eV along Path I in HfZrTe_4_. The flexible Te‐5*p* orbitals probably work better because they can more easily accommodate Na^+^ larger ionic radius.

The path‐dependent study shows that Path I is the main pathway for Na^+^ movement, but the other paths remain open and have barriers of less than 0.151 eV. This is important for SIBs that need to handle high current. HfZrTe_4_ has the lowest barriers for Li^+^ (0.206 eV) and Na^+^ (0.046 eV) migration. HfZrTe_4_ also has the least barrier anisotropy (38%), indicating that its transport is more isotropic than that of the other compositions. These basic ideas about how ions move through materials are significant for making electrode materials that work well. For instance, designing electrode architectures to favor Path I diffusion could make both Li^+^‐ion and Na^+^‐ion diffusion faster.

Short‐chain Li_2_S and Na_2_S molecules were also examined. Figure 4b presents decomposition barriers for Li_2_S and Na_2_S on the HfTiTe_4_, ZrTiTe_4_, and HfZrTe_4_ surfaces, revealing differences in stability and reaction pathways. For Li_2_S, the barriers start from zero for the initial state and increase progressively, reaching 1.02 eV on HfTiTe_4_, 0.96 eV on ZrTiTe_4_, and 0.87 eV on HfZrTe_4_ at the peak step. The highest barrier is observed for HfTiTe_4_, indicating greater stability of Li_2_S species against decomposition, while HfZrTe_4_ exhibits the lowest barrier, which could facilitate faster conversion during cycling. For Na_2_S, a similar trend is observed, though the barriers are generally lower than in the Li_2_S case at most steps, reflecting the weaker interaction of Na‐based species with the substrates. The maximum barriers for Na_2_S are 0.92 eV for HfTiTe_4_, 0.89 eV for ZrTiTe_4_, and 0.84 eV for HfZrTe_4_. These lower activation energies suggest that Na_2_S is more prone to decomposition, which may enhance kinetics but could also risk polysulfide loss if not adequately anchored. Overall, the data indicate that HfTiTe_4_ shows the best resistance to Li_2_S and Na_2_S decomposition, and ZrTiTe_4_ has a good balance of stability and kinetics. At the same time, HfZrTe_4_ decomposes more rapidly, making it potentially advantageous for high‐rate applications.

### 3.5 Ion‐Storage Capacity and Average Open‐Circuit Voltage

The performance of the electrode material was analyzed by evaluating the ion‐storage capacity and open‐circuit voltage (OCV).^[^ ^61^ ^]^ The topological materials HfTiTe_4_, ZrTiTe_4_, and HfZrTe_4_ with the stoichiometry of ABTe_4_ exhibit the capability to store Li^+^/Na^+^ ions. Figure 4c depicts the adsorption of ions in a 3 × 3 × 1 supercell to progressively enhance ion counts in ABTe_4_ while considering seven distinct concentrations of metal ion (M*_x_* = 0.01, 0.5, 1.5, 2.0, 2.5, and 3.0). It is noted that the binding energy of Li^+^/Na^+^ ions declines marginally as their concentration increases, mainly due to repulsive interactions between adjacent atoms. The DOS and PDOS results for the ABTe_4_ monolayer fully intercalated with Li^+^/Na^+^ ions show increased electronic states near the Fermi level, indicating enhanced electrical conductivity (Figures S20 and S21, Supporting Information). The storage capacities (*C*) of batteries are estimated using the following equation:

(9) $$C=\frac{ZxF}{3.6M}\;$$

where *Z* indicates the valence number, with a value of 1 for Li^+^ and Na^+^ ions; *x* is the maximum concentration of Li^+^/Na^+^ ions in the ABTe_4_ monolayer; F is the Faraday constant (96 485.3329 C mol^‒1^); and M represents the molar mass of pristine ABTe_4_. These HfTiTe_4_, ZrTiTe_4_, and HfZrTe_4_ anodes show the ultrahigh specific capacities of over 1600, 1350, and 1200 mAh g^‒1^, respectively, while storing monovalent ions. Capacity increases uniformly with ion concentration, reaching 1640, 1203, and 1343 mAh g^‒1^ at the maximum coverage of Li^+^ ions, while 1366, 1002, and 1119 mAh g^‒1^ for Na^+^ ions. At a converge M*_x_* = 0.01, the Li^+^‐ion binding energy starts at −1.746, −1.659, and −1.490 eV. It then slowly drops to −0.471, −0.409, and −0.539 eV at saturation. For Na^+^ ions, the binding energies start from −1.671, −1.648, and −1.565 eV. At extreme coverage, the energy drops to −0.353, −0.340, and −0.416 eV for HfTiTe_4_, ZrTiTe_4_, and HfZrTe_4_. Meanwhile, the lattice parameters change by only ≈1.1% upon Li intercalation and ≈1.5% upon Na intercalation, indicating minimal structural swelling. Primarily due to weaker ion surface interactions at high occupancy and growing electrostatic repulsion between neighboring ions. **Table** **1** shows that our studied monolayers exhibit comparative capacity as Net W (≈ 1675 mAh g^‒1^ for Li^+^ ion) and higher than ψ‐graphene (624 mAh g^‒1^ for Li^+^ ion), Θ‐graphene (876 mAh g^‒1^ for Li^+^ ion and 1275 mAh g^‒1^ for Na^+^ ion), and Net‐τ (588 mAh g^‒1^ for Li^+^ ion). Evaluating the voltage profile is crucial for determining the high performance of the rechargeable battery. We have computed the average voltage as a function of metal atom concentration by varying *x* in the ABTe_4_ monolayer. The average OCV for the 2D monolayer is determined using the following formula:

(10) $$\mathrm{ABT}e_{4}+xM\to M_{x}\mathrm{ABT}e_{4}$$

(11) $$\mathrm{OCV}\;=-\frac{E_{M_{x}\mathrm{ABT}e_{4}}-E_{\mathrm{ABT}e_{4}}-xE_{M}}{xe}$$

**Table 1:** Electrochemical performance comparison of 2D topological TMC materials with previously reported 2D materials for Li^+^ and Na^+^‐ion batteries in terms of theoretical capacities (in mAh g^‒1^), diffusion barriers (in eV), average OCV (in V), and conductivity. (–) shows no data available.

Materials	Capacity		Diffusion barrier		Average OCV		Electronic band
	Li^+^	Na^+^	Li^+^	Na^+^	Li^+^	Na^+^	
Graphite	372	< 35	0.40	0.30	0.11	–	Metallic
HfTiTe_4_^(This Work)^	≈ 1640	≈ 1366	0.220	0.116	≈ 0.47	≈ 0.35	Topological metal
ZrTiTe_4_^(This Work)^	≈ 1203	≈ 1202	0.214	0.110	≈ 0.40	≈ 0.34	Topological metal
HfZrTe_4_^(This Work)^	≈ 1343	≈ 1119	0.205	0.09	≈ 0.53	≈ 0.41	Topological metal
Ti_2_PTe_2_^[^ ^62^ ^]^	830	421	0.20	0.090	0.7	0.20	Metallic
V_2_BTe_2_^[^ ^63^ ^]^	–	290	–	0.097	–	0.163	Metallic
Ti_2_BTe_2_^[^ ^64^ ^]^	296	296	0.19	0.08	0.06	0.03	Metallic
Popgraphene^[^ ^65^ ^]^	1487	–	< 0.55	–	0.45	–	Metallic
Phagraphene^[^ ^66^ ^]^	< 372	–	0.42	–	–	–	Metallic
Biphenylene^[^ ^66^ ^]^	< 372	–	0.68	–	–	–	Metallic
Net W^[^ ^67^ ^]^	1675	–	0.40	–	0.42	–	Metallic
Net Y^[^ ^68^ ^]^	–	1787	–	0.35	–	0.44	Metallic
ψ‐graphene^[^ ^69^ ^]^	624	–	0.31	–	0.01	–	Metallic
Θ‐graphene^[^ ^70^ ^]^	876	1275	≤ 0.5	≤ 0.5	≤ 0.6	≤ 0.6	Semiconducting
Net‐τ^[^ ^71^ ^]^	588	–	0.29	–	0.004	–	Metallic
Porous graphene^[^ ^72^ ^]^	2857	–	0.37	–	–	–	Semiconducting
Graphenylene^[^ ^73^ ^]^	930	–	0.99	–	–	–	Metallic
GDY^[^ ^74^ ^]^	624	–	0.18	–	–	–	Semiconducting
Carbon ene‐yne^[^ ^75^ ^]^	2680	1788	0.60	0.58	–	–	Metallic
C_18_‐sheet^[^ ^76^ ^]^	–	991	–	0.46	–	0.12	Metallic
Boron‐GDY^[^ ^77^ ^]^	1294	1617	0.36	0.28	–	0.14	Semiconducting
B_7_P_2_^[^ ^78^ ^]^	3117	3117	0.59	0.32	0.25	0.20	Metallic

The theoretical average voltage profiles as a function of concentration *x* for Li^+^/Na^+^ ions in 2D HfTiTe_4_, ZrTiTe_4_, and HfZrTe_4_ are shown in Figure 4d. The OCV exhibits a similar decline with increasing ion coverage, mirroring the trend in binding energies. The initial OCV values for Li^+^ ions are 1.746 V (HfTiTe_4_), 1.659 V (ZrTiTe_4_), and 1.490 V (HfZrTe_4_). At complete saturation, these values drop to 0.47–0.54 V. Na^+^‐ion OCVs show a similar pattern, starting at 1.671, 1.648, and 1.565 V and dropping to ≈0.34–0.42 V at full load. While comparing (Table 1), the OCVs of HfTiTe_4_, ZrTiTe_4_, and HfZrTe_4_ are higher than C_18_‐sheet (V = 0.12 V for Na^+^), Boron‐GDY (V = 0.14 V for Na^+^), and B_7_P_2_ (V = 0.25 V for Li^+^ and and V = 0.20 V for Na^+^). However, the OCVs of HfZrTe_4_ are comparable to 3D monosilicene (V = 0.83 V for Li^+^) and 3D ortho‐silicene (V = 0.39 V for Na^+^). Overall, HfTiTe_4_ has strong Li^+^/Na^+^ binding and high initial voltages, enabling rapid ion capture during early stages of cycling. ZrTiTe_4_ maintains relatively stable voltage over a wide range of coverages, whereas HfZrTe_4_ balances intermediate binding energies with high capacity, making it suitable for high‐energy‐density applications.

**Figure 5:**
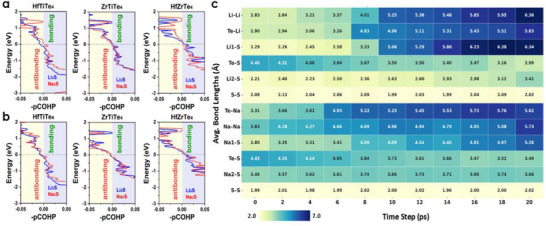
Stability comparisons of HfTiTe_4_, ZrTiTe_4_, and HfZrTe_4_ with Li_2_S and Na_2_S. a) pCOHP analysis of HfTiTe_4_, ZrTiTe_4_, and HfZrTe_4_ with Li_2_S (blue) and Na_2_S (red), showing average metal–Te bond responses. Bonding (yellow) and antibonding (blue) regions indicate that Na_2_S generally enhances stability relative to Li_2_S. b) ‐pCOHP analysis of of Li_2_S (blue) and Na_2_S (red) adsorption on HfTiTe_4_, ZrTiTe_4_, and HfZrTe_4_ monolayers. HfZrTe_4_ has strongest bonding (yellow region) and minimal antibonding (blue region) at the Fermi level. In contrast, HfTiTe_4_ shows stronger antibonding with Li_2_S, and ZrTiTe_4_ shows moderate stability. c) Heatmap of bond distances (Å) in HfZrTe_4_–Li_2_S/Na_2_S clusters during 20 ps AIMD at 300 K. The dynamics reveal strong initial alkali–sulfur coordination, gradual alkali detachment, and persistent S═S bonding, highlighting adaptability for polysulfide anchoring.

This trend indicates that all three hosts can accommodate multiple ions while maintaining structural stability. These compositional variations in telluride monolayers directly influence binding strength, voltage stability, and the maximum achievable capacity, enabling targeted optimization for the next‐generation Li^+^/Na^+^ batteries.^[^ ^79^ ^]^ To further elucidate these effects, the projected crystal orbital Hamilton population (pCOHP) analysis offers comprehensive insights into the bond stability of HfTiTe_4_, ZrTiTe_4_, and HfZrTe_4_ monolayers upon the adsorption of Li_2_S and Na_2_S, highlighting significant variations in interfacial behavior (**Figure** **5**a). When Li_2_S is adsorbed onto HfTiTe_4_, it creates strong antibonding states around the Fermi level. Weakening the Hf–Te and Ti–Te bonds destabilizes the structure. However, Na_2_S adsorption reduces antibonding, making Na_2_S–HfTiTe_4_ more stable at the interface. Adsorption of Li_2_S and Na_2_S by ZrTiTe_4_ and HfZrTe_4_ maintains strong metal–Te bonding with low antibonding states, confirming stable lattice frameworks. Polysulfide adsorption has little effect on strong covalent metal–Te interactions, ensuring stability. The blue curves indicate how the average metal–Te bond responds to Li_2_S adsorption, and the red curves show how it responds to Na_2_S. In both systems, Na_2_S makes fewer antibonding contributions, so the structure is better preserved during the cycle. The bonding tendencies for Li_2_S and Na_2_S systems are more evident when analyzing the key atomic contacts shown in Figure 5b. For Li_2_S systems, these contacts include Li–Li, Li–S, S–S, Li–Te, and S–Te. For Na_2_S systems, they consist of Na–Na, Na–S, S–S, and S–Te. HfTiTe_4_ destabilizes Li_2_S because of robust antibonding near the Fermi level, whereas Na_2_S remains stable. ZrTiTe_4_ has only weak antibonding at lower energies, which means that both polysulfides will be better anchored. In HfZrTe_4_, the pCOHP curves reveal strong bonding and very little antibonding near the Fermi level. This means that the interfacial structure is the most stable of the three. This bonding hierarchy corresponds to electrochemical behavior: stronger bonding results in better polysulfide immobilization, reduced shuttle effect, and increased cycling stability.

To assess the dynamic evolution of these interactions, AIMD simulations were conducted on Li_2_S and Na_2_S clusters adsorbed on HfZrTe_4_ during a 20 ps trajectory at 300 K (Figure S22, Supporting Information). Heatmaps of bond distances (Figure 5c) show that Li═Li and Na═Na bonds get a lot longer over time — Li═Li goes from 2.83 Å to ≈6.34 Å and Na═Na goes from 3.83 Å to more than 5.7 Å. This shows that ions are moving and partially breaking apart. Li═S bonds are strong at first (≈2.29 Å), but they get longer than 6.2 Å. Na═S bonds, on the other hand, range from 2.8 to 3.7 Å, indicating that sodium has weaker ionic interactions. Te═Li and Te═Na bonds also get longer with time, which means that direct ion‐lattice coupling is less intense as the temperature changes. The S═S bonds, on the other hand, remain stable at roughly 2.0–2.1 Å, indicating that the sulfur backbone remains intact even as alkali ions move around. Over time, Te═S bonds get shorter, from ≈4.4 to ≈3.0 Å. This suggests that the lattice is reorganized, bringing sulfur closer together and making the structure more stable. These results show that HfZrTe_4_ has a good balance of strong initial alkali‐sulfur binding, which is essential for immobilizing polysulfides in the early stages, and enough bond flexibility to allow ions to move around during cycling. Na_2_S consistently produces fewer destabilizing antibonding states than Li_2_S, suggesting that systems based on Na may be better at maintaining lattice stability over time. HfZrTe_4_ is an emerging high‐performance sulfur host for both Li═S and Na═S batteries because it exhibits strong bonding, reduced antibonding near the Fermi level, and an adaptable lattice response. It can stop the shuttle effect while supporting rapid ion mobility. These results show that strong initial chemisorption, fractional antibonding around the Fermi level, and adaptive lattice rearrangement are essential for mechanical and electrochemical performance. HfZrTe_4_ offers a solution to the stability‐kinetics conflict in sulfur hosts by allowing polysulfide immobilization and ion migration. Na_2_S systems exhibit greater stability, suggesting longer cycle life and rate capability. This combination of bonding stability, structural flexibility, and electrochemical efficiency makes topological telluride monolayers promising multifunctional platforms for next‐generation Li═S and Na═S batteries.

## 4 Conclusion

This study establishes that topological HfTiTe_4_, ZrTiTe_4_, and HfZrTe_4_ monolayers act as truly dual‐function electrodes, capable of operating both as ultrafast‐charging anodes and high‐activity sulfur hosts. They have record‐high theoretical capacities of ≈1600 mAh g^−1^ for Li^+^ and ≈1350 mAh g^−1^ for Na^+^. These capacities are accompanied by exceptionally low diffusion barriers, specifically < 0.22 eV for Li^+^ and < 0.12 eV for Na^+^. Additionally, the OCVs are in the range of 0.47–0.54 V for Li⁺ and 0.34–0.42 V for Na^+^. These metrics significantly surpass those of state‐of‐the‐art 2D carbons and phosphorene analogues. The unique topological surface states enable strong electronic coupling with polysulfides, leading to *i*) robust anchoring energies that exceed the solvation energies in DME/DOL electrolytes, thereby suppressing the shuttle effect, and *ii*) catalytic promotion of the sulfur‐reduction sequence with a minimal Gibbs free‐energy penalty of 0.934 eV for Li_2_S_4_ → Li_2_S_2_ on HfZrTe_4_. HfZrTe_4_ is particularly suited for fast‐charging devices owing to its low thermodynamic overpotential, minimum kinetic barriers, and nearly isotropic ion diffusion. AIMD simulations confirm that the telluride frameworks remain dynamically and thermally stable up to 500 K, providing reliable volume buffering and continuous electron delocalization during both Li^+^/Na^+^ ions insertion and sulfur conversion. These results highlight the potential to integrate quantum‐material properties with electrochemical functionality to develop high‐energy, high‐rate rechargeable batteries based on topological ternary tellurides.

## Conflict of Interest

The authors declare no conflict of interest.

## Supporting information



Supporting Information

## Data Availability

The data that support the findings of this study are available in the supplementary material of this article.
