# Supplementary material for: Ultra‐Stable Topological Telluride Monolayers for Next‐Generation Battery Anodes and Sulfur Hosts
Source: Adv Sci (Weinh). 2025 Nov 3;13(4):e15841. doi: 10.1002/advs.202515841 (PMC12822415; doi:10.1002/advs.202515841)
Supplement: Supplementary file 1 — Supporting Information [file ADVS-13-e15841-s001.docx]

**Supporting Information**

**Ultra-Stable Topological Telluride Monolayers for Next-Generation Battery Anodes and Sulfur Hosts**

Shehzad Ahmed^1,^*, Awais Ghani^2^, Rashid Mehmood^3^, Ahsan Ali^4^, Naveed Hussain^5^, Abdul Khaliq^4^, Jun Han^6,7^, Kemeng Ji^6,7,^*, Danish Khan^8,^*, and Imran Muhammad^7,^*

^1^ *China-UK Low Carbon College, Shanghai Jiao Tong University, Shanghai, 201306, China.*

^2^ *Smart Materials for Architecture Research Lab, Innovation Center of Yangtze River Delta, Zhejiang University, Jiaxing 314100, China.*

^3^ *Department of Engineering Materials, Engineering School of São Carlos, University of São Paulo, Avenue João Dagnone, 1100, São Carlos 13563-120, Brazil.*

^4^ *Department of Physics, Guangdong Technion Israel Institute of Technology, Shantou, 515063, Guangdong, PR China.*

^5^ *Department of Electrical Engineering and Computer Science, University of California Irvine, Irvine, CA, 92697 USA.*

^6^ *Key Laboratory for Green Chemical Technology of Ministry of Education, Collaborative Innovation Center of Chemical Science and Engineering (Tianjin), School of Chemical Engineering and Technology, Tianjin University, Tianjin 300350, P. R. China.*

^7^ *National Industry-Education Platform of Energy Storage, Tianjin University, 135 Yaguan Road, Tianjin, 300350, P.R. China.*

^8^ *College of New Materials and New Energies, Shenzhen Technology University, Shenzhen 518118, Guangdong, China.*

**CONTENTS**

|  | Potential energy fluctuations and corresponding geometries during molecular-dynamics simulations for 300 and 500 K | Figures S1‒S4 |
| --- | --- | --- |
|  | Partial densities of states of pure and single metal-ion adsorbed HfTiTe_4_, ZrTiTe_4_, and HfZrTe_4_ | **Figures S5‒S7** |
|  | Bonding analysis of ABX_4_ monolayers during alkali-ion intercalation | **Figure S8** |
|  | Partial densities of states of Li_2_S, Li_2_S_2_, Li_2_S_4_, Li_2_S_6_, Li_2_S_8_, and S_8_ adsorbed HfTiTe_4_, ZrTiTe_4_, and HfZrTe_4_ substrates | **Figures S9‒S13** |
|  | Geometries and Partial densities of states of Na_2_S, Na_2_S_2_, Na_2_S_4_, Na_2_S_6_, Na_2_S_8_, and S_8_ adsorbed HfTiTe_4_, ZrTiTe_4_, and HfZrTe_4_ substrates | **Figures S14‒S16** |
|  | Li_2_S*_n_* and Na_2_S*_n_* species response to HfZrS_4_ and HfZrSe_4_ | **Figures S17‒S19** |
|  | Partial densities of states of dense metal-ion adsorbed HfTiTe_4_, ZrTiTe_4_, and HfZrTe_4_ | **Figures S20 and S21** |
|  | AIMD simulations for Li_2_S and Na_2_S clusters response on monolayer HfZrTe_4_ at 300 K for 20 ps | **Figure S22** |

1. **Potential energy fluctuations and corresponding geometries during molecular-dynamics simulations for 300 and 500 K.**


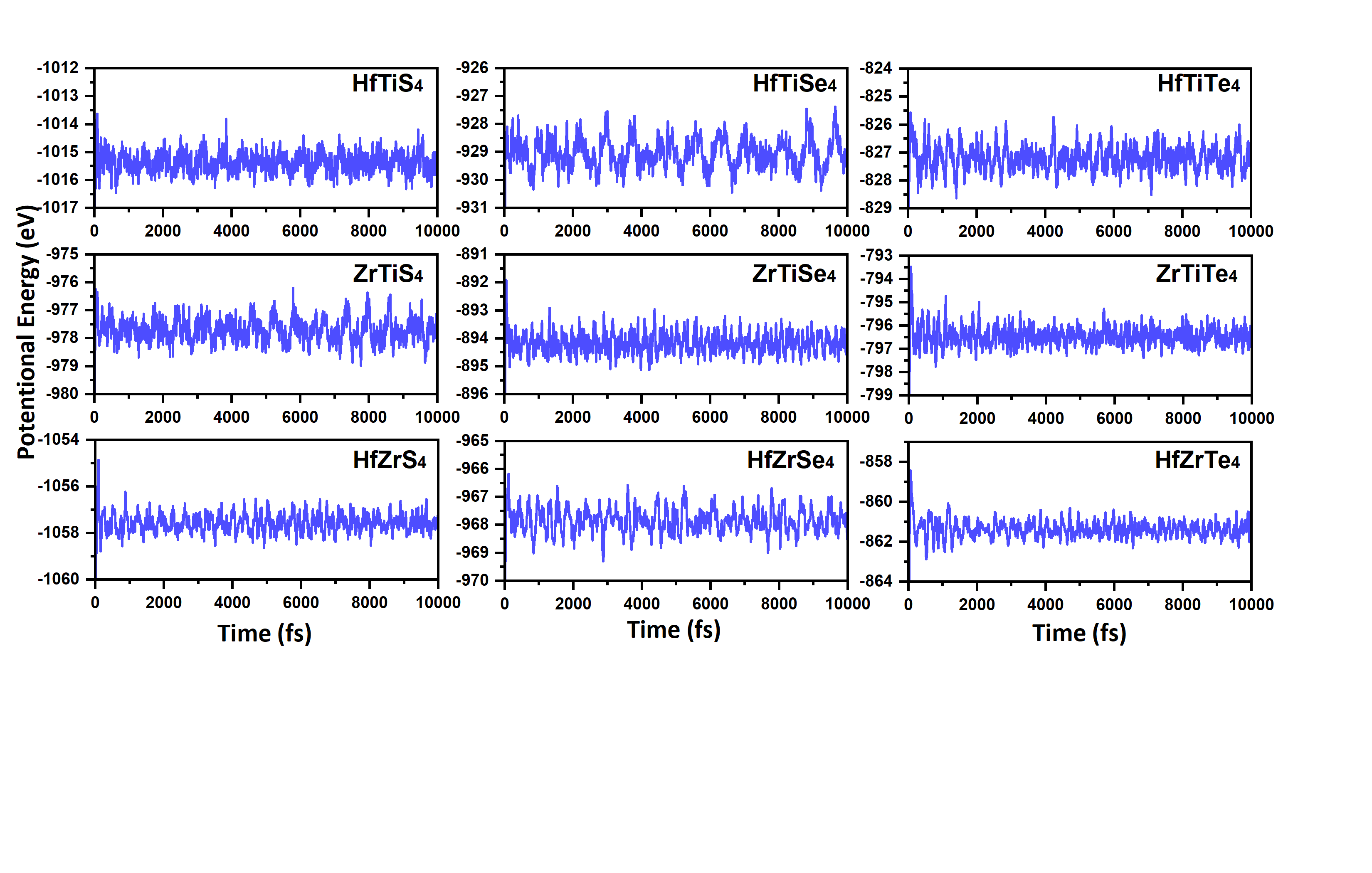


**Figure S1.** The fluctuations of potential energy during the AIMD simulations for 10 psec at 300 K of nine distinct ternary combinations of monolayer TTMCs with a chemical formula ABX_4_, where AB includes Hf, Zr, and Ti, while X represents S, Se, and Te.

**
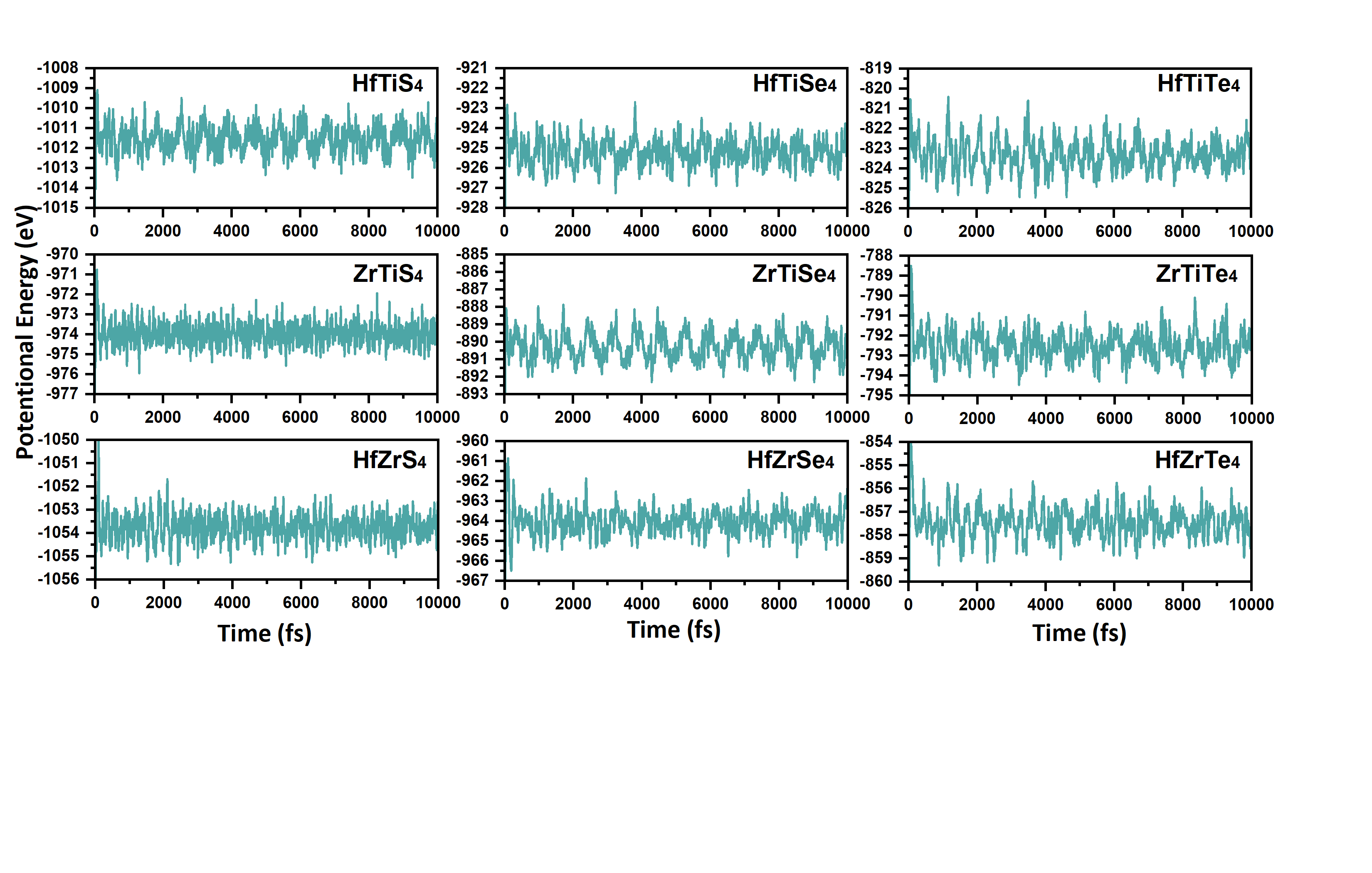
**

**Figure S2.** The fluctuations of potential energy during the AIMD simulations for 10 psec at 500 K of nine distinct ternary combinations of monolayer TTMCs with a chemical formula ABX_4_, where AB includes Hf, Zr, and Ti, while X represents S, Se, and Te.


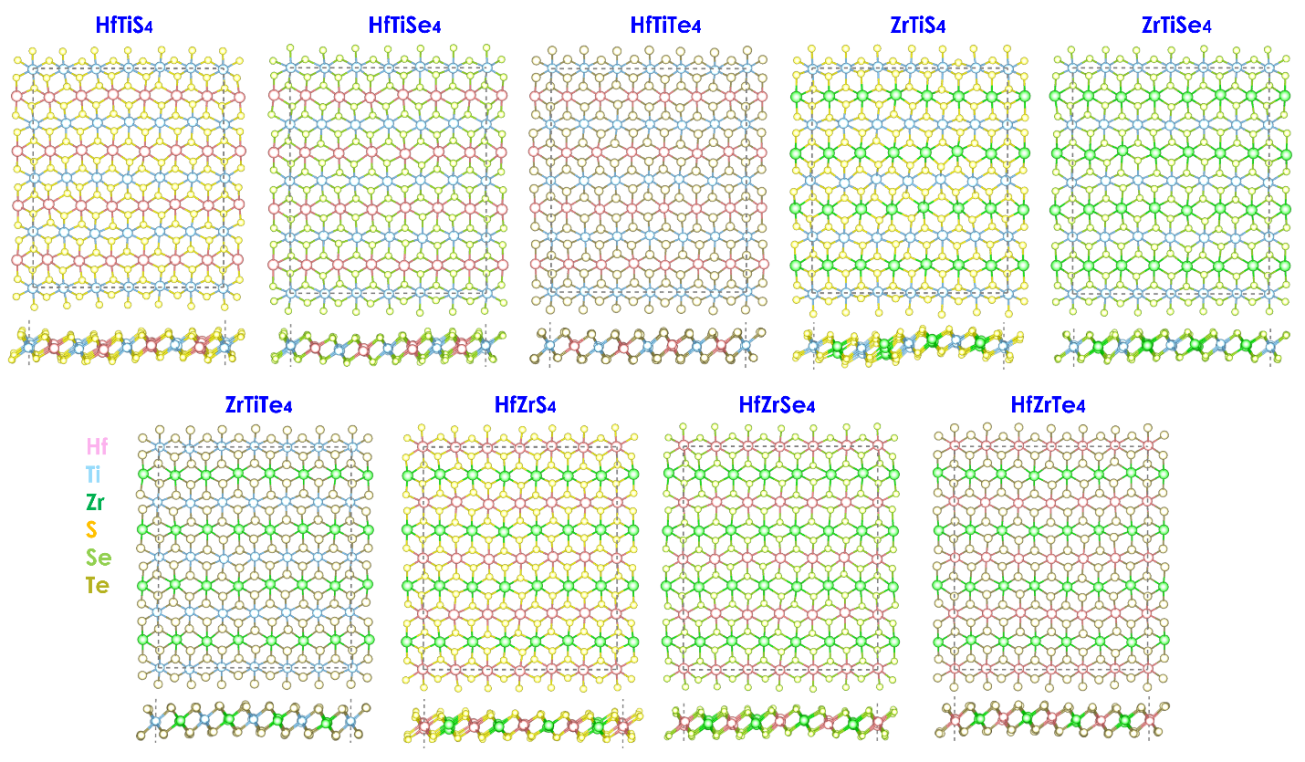


**Figure S3.** Top view and side view of atomic structures of nine distinct ternary combinations of monolayer TTMCs with a chemical formula ABX_4_ (AB represents Hf, Zr, and Ti and X represents S, Se, and Te) after the AIMD simulations for 10 psec at 300 K**.**


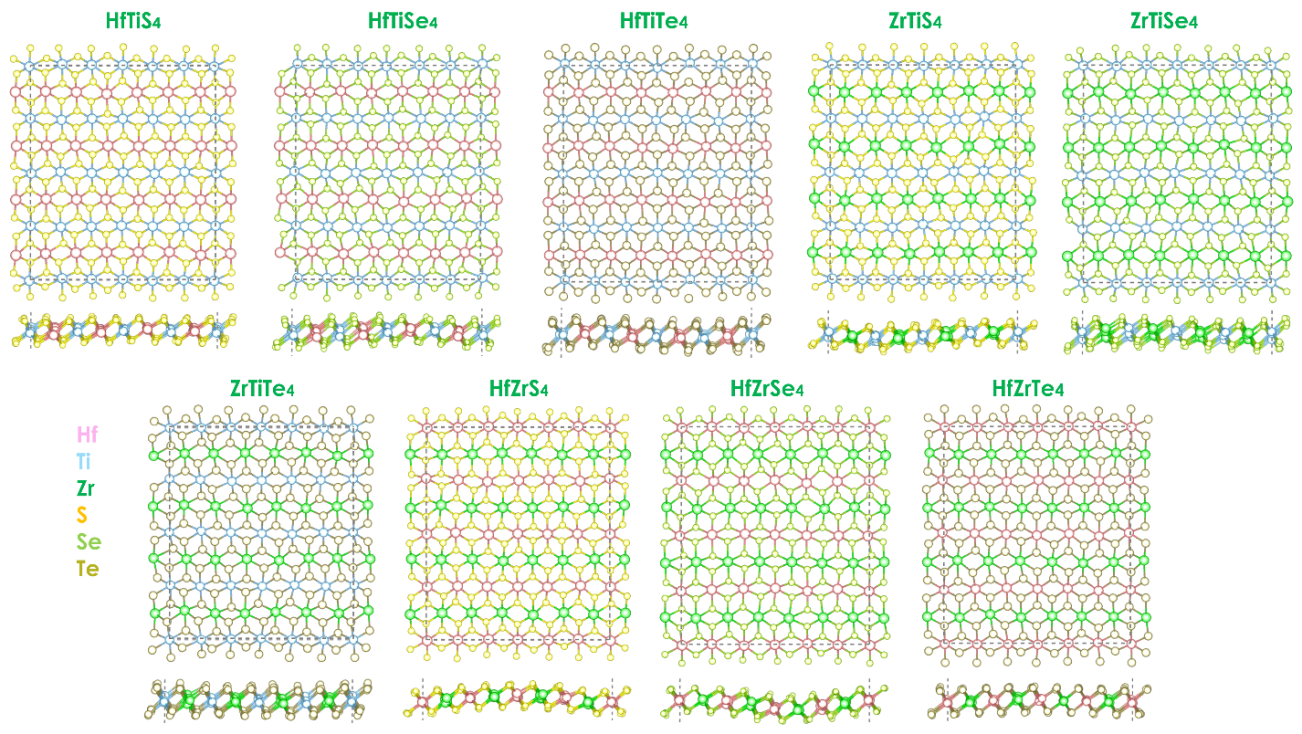


**Figure S4.** Top view and side view of atomic structures of nine distinct ternary combinations of monolayer TTMCs with a chemical formula ABX_4_ (AB represents Hf, Zr, and Ti and X represents S, Se, and Te) after the AIMD simulations for 10 ps at 500 K.

1. **Partial densities of states of pure and single metal-ion adsorbed HfTiTe_4_, HfZrTe_4_, and ZrTiTe_4_.**


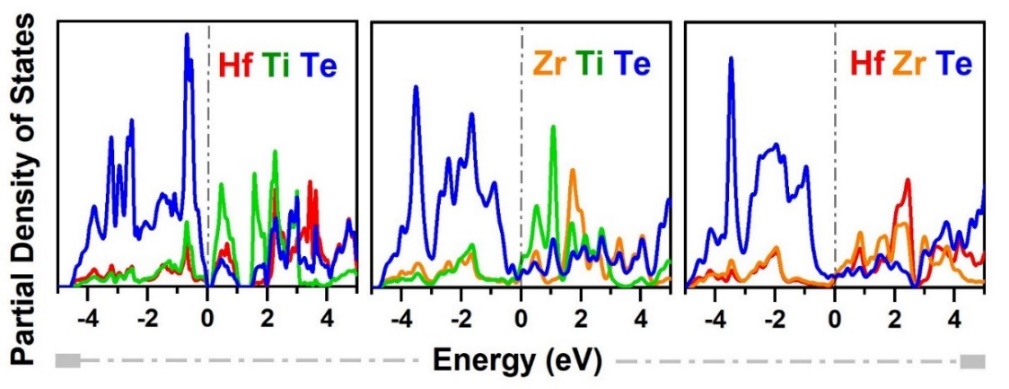


**Figure S5.** Partial densities of states of **HfTiTe₄, ZrTiTe₄, and HfZrTe₄ monolayers.**


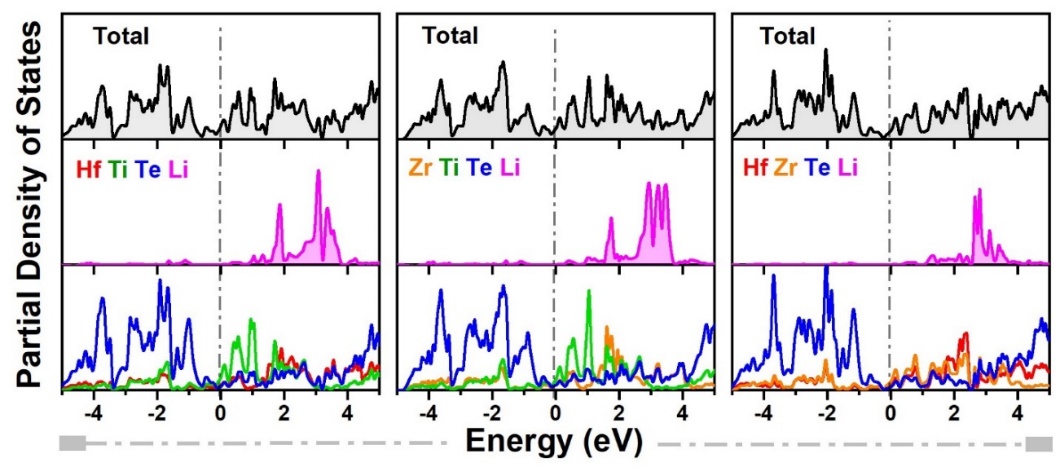


**Figure S6.** Partial densities of states of single lithium-ion adsorbed **HfTiTe₄, ZrTiTe₄, and HfZrTe₄ monolayers.**


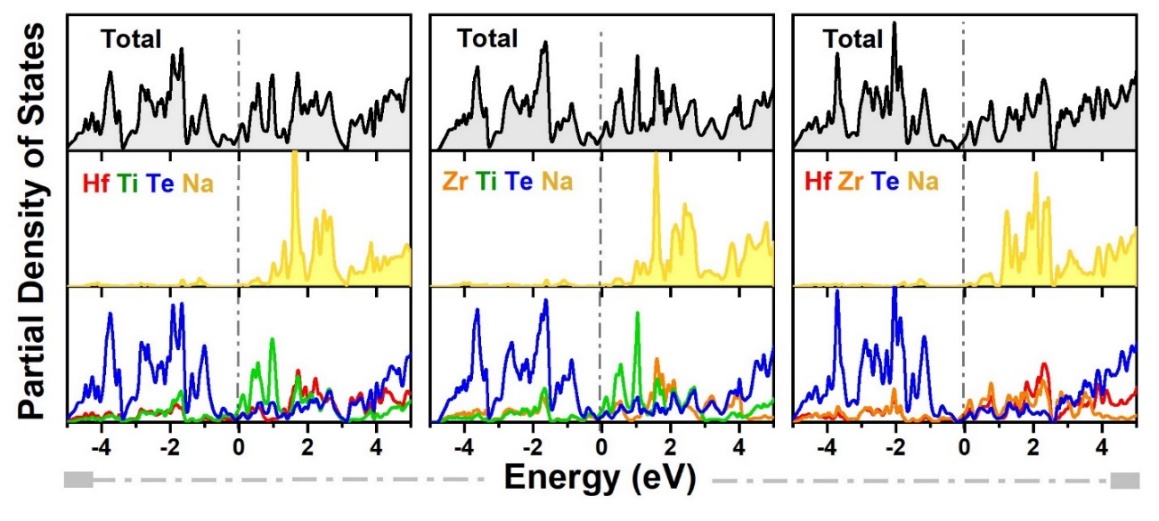


**Figure S7.** Partial densities of states of single sodium-ion adsorbed **HfTiTe₄, ZrTiTe₄, and HfZrTe₄ monolayers.**

1. **Bonding analysis of ABX_4_ monolayers during alkali-ion intercalation.**


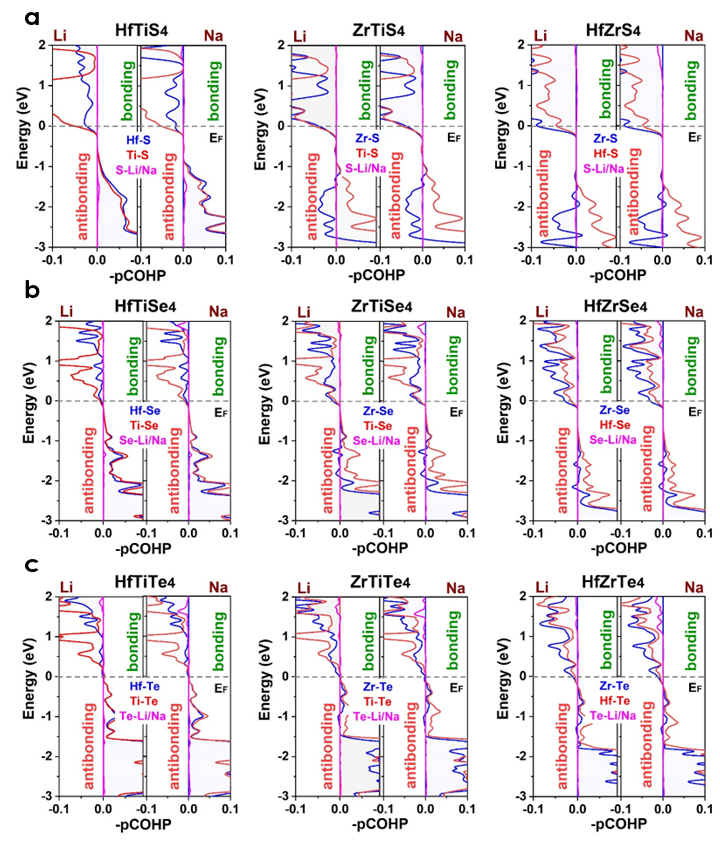


**Figure S8.** Bonding analysis of ABX_4_ monolayers during alkali-ion intercalation. Projected crystal orbital Hamiltonian population (pCOHP) plots for (a) HfTiS₄, ZrTiS₄, and HfZrS₄, (b) HfTiSe_4_, ZrTiSe_4_, and HfZrSe_4_ monolayers, and (c) HfTiTe_4_, ZrTiTe_4_, and HfZrTe_4_ **monolayers upon Li/Na ion adsorption.**

1. **Geometries and states of Li_2_S, Li_2_S_2_, Li_2_S_4_, Li_2_S_6_, Li_2_S_8_, and S_8_ adsorbed HfTiTe_4_, HfZrTe_4_, and ZrTiTe_4_ substrates.**


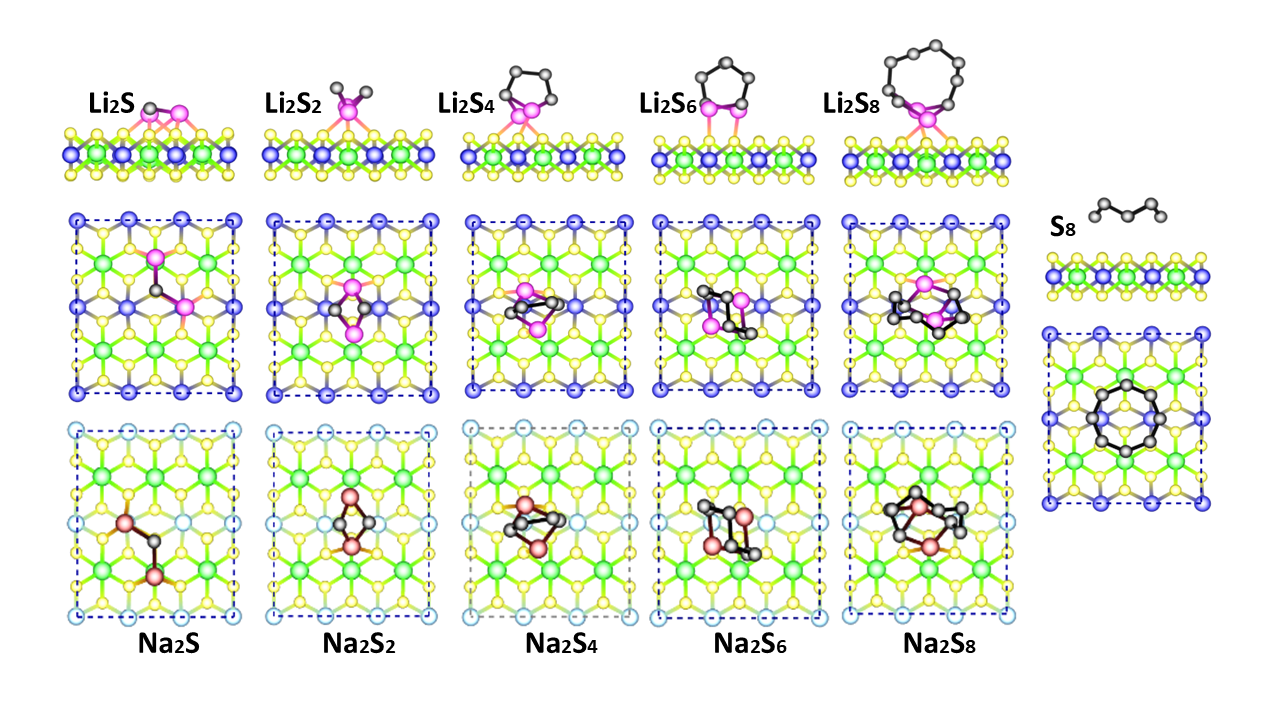


**Figure S9.** The optimized structures of ABTe_4_ **monolayers upon** Li_2_S_n_/Na_2_S_n_ (n = 1, 2, 4, 6, and 8) and S_8_ clusters **adsorption**.


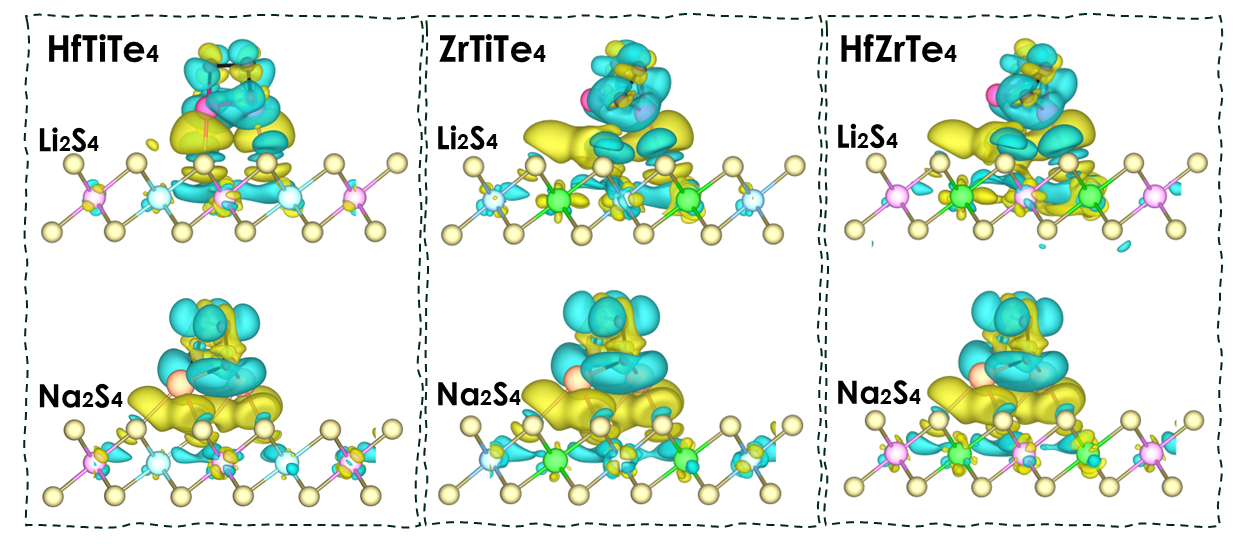


**Figure S10.** Charge–density difference isosurfaces for Li_2_S_4_ (top) and Na₂S_4_ (bottom) adsorbed on HfTiTe_4_, ZrTiTe_4_, and HfZrTe_4_. Yellow and cyan denote charge accumulation and depletion, respectively, revealing stronger interfacial polarization for Li_2_S_4_/Na_2_S_4_ and distinct metal–chalcogen participation across the three hosts. Atoms: Te (gold), Ti (light blue), Hf/Zr (pink/green).


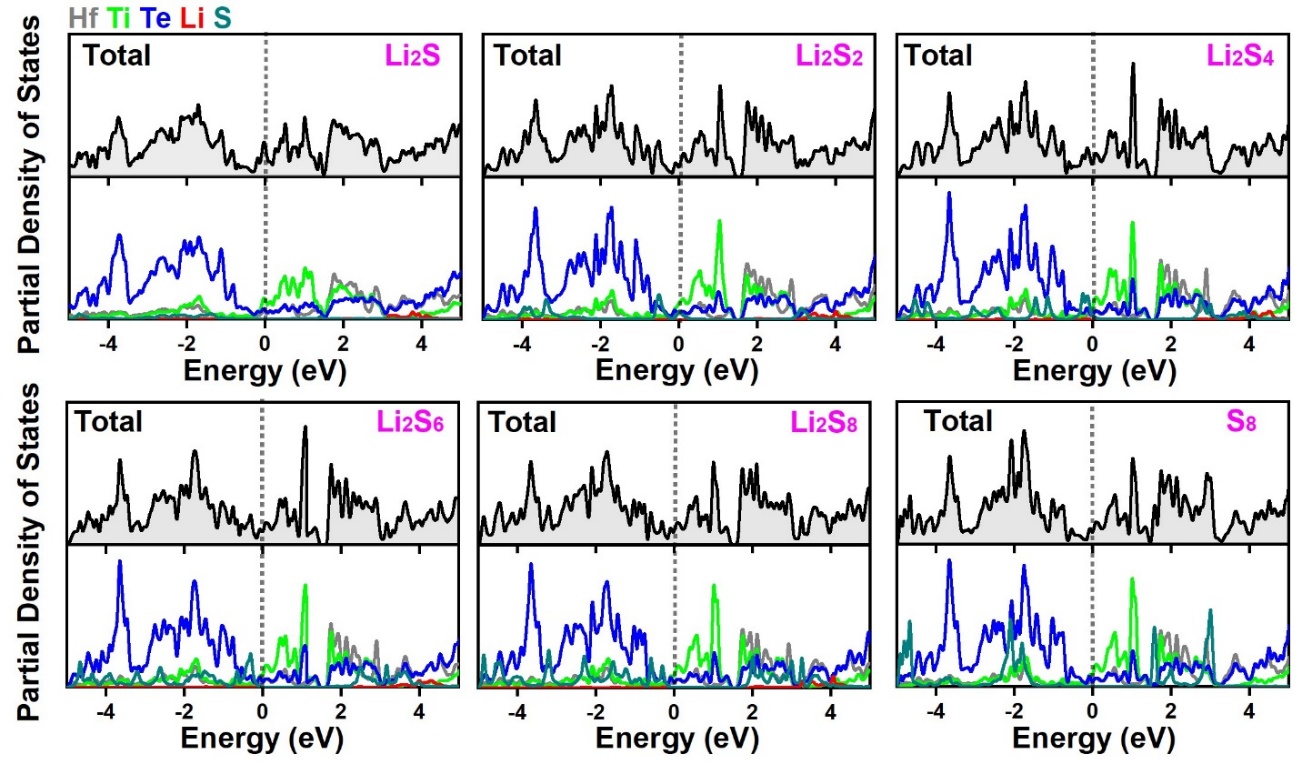


**Figure S11.** Partial densities of states of Li_2_S, Li_2_S_2_, Li_2_S_4_, Li_2_S_6_, Li_2_S_8_, and S_8_ adsorbed HfTiTe_4_ substrate.


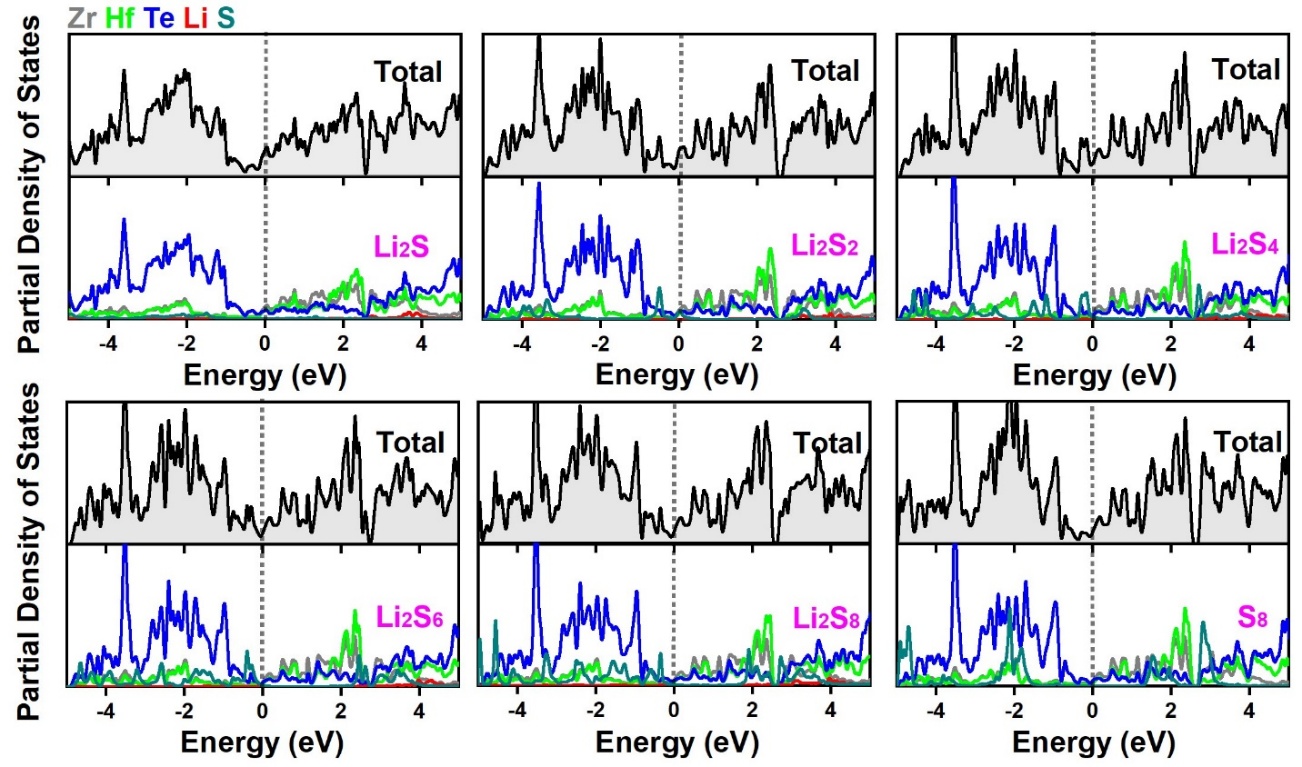


**Figure S12.** Partial densities of states of Li_2_S, Li_2_S_2_, Li_2_S_4_, Li_2_S_6_, Li_2_S_8_, and S_8_ adsorbed HfZrTe_4_ substrate.


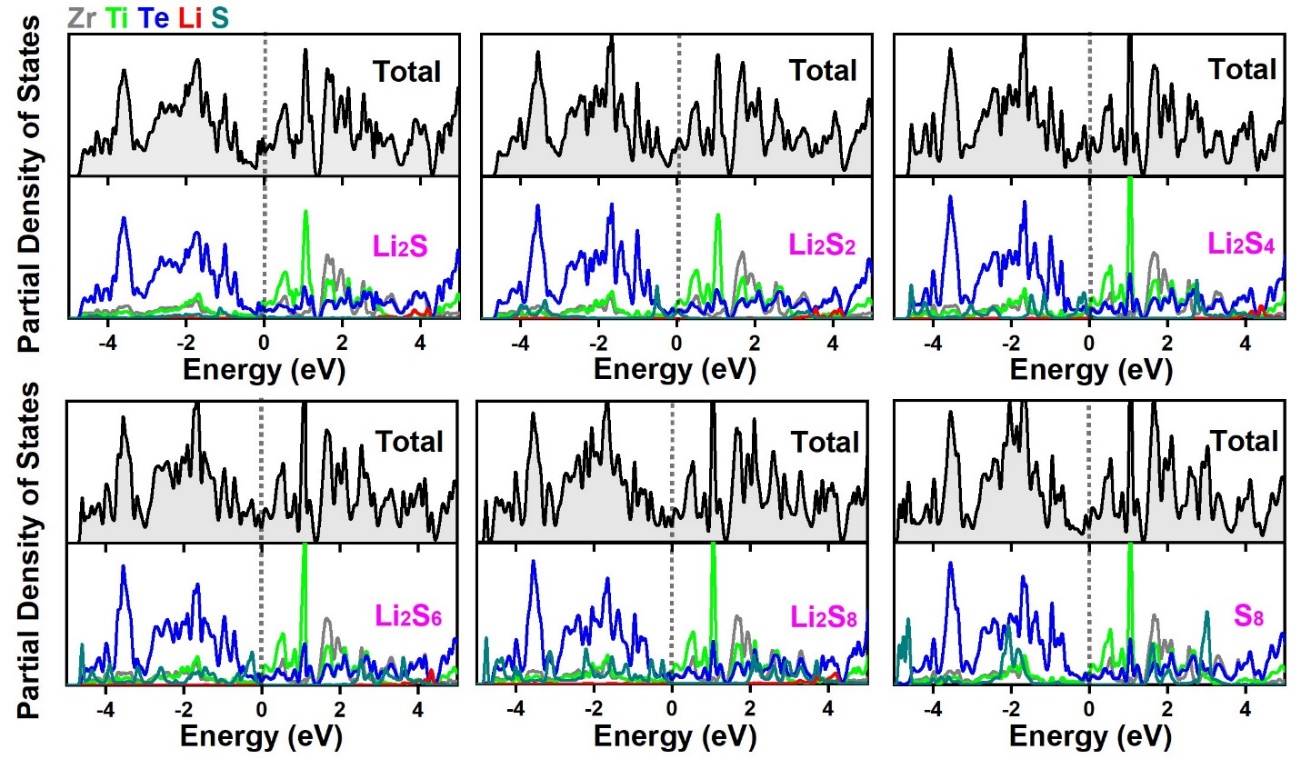


**Figure S13.** Partial densities of states of Li_2_S, Li_2_S_2_, Li_2_S_4_, Li_2_S_6_, Li_2_S_8_, and S_8_ adsorbed ZrTiTe_4_ substrate.

1. **Partial densities of states of Na_2_S, Na_2_S_2_, Na_2_S_4_, Na_2_S_6_, and Na_2_S_8_ adsorbed HfTiTe_4_, HfZrTe_4_, and ZrTiTe_4_ substrates.**


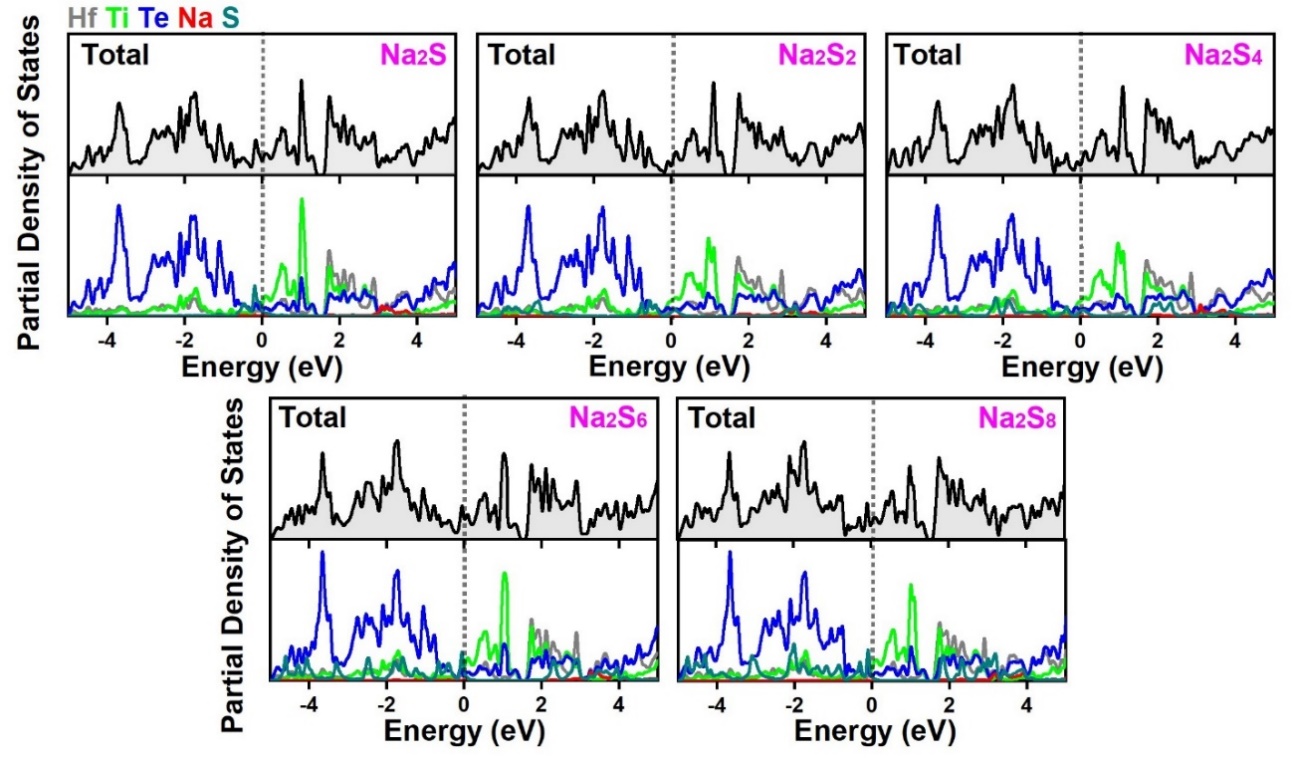


**Figure S14.** Partial densities of states of Na_2_S, Na_2_S_2_, Na_2_S_4_, Na_2_S_6_, and Na_2_S_8_ adsorbed HfTiTe_4_ substrate.


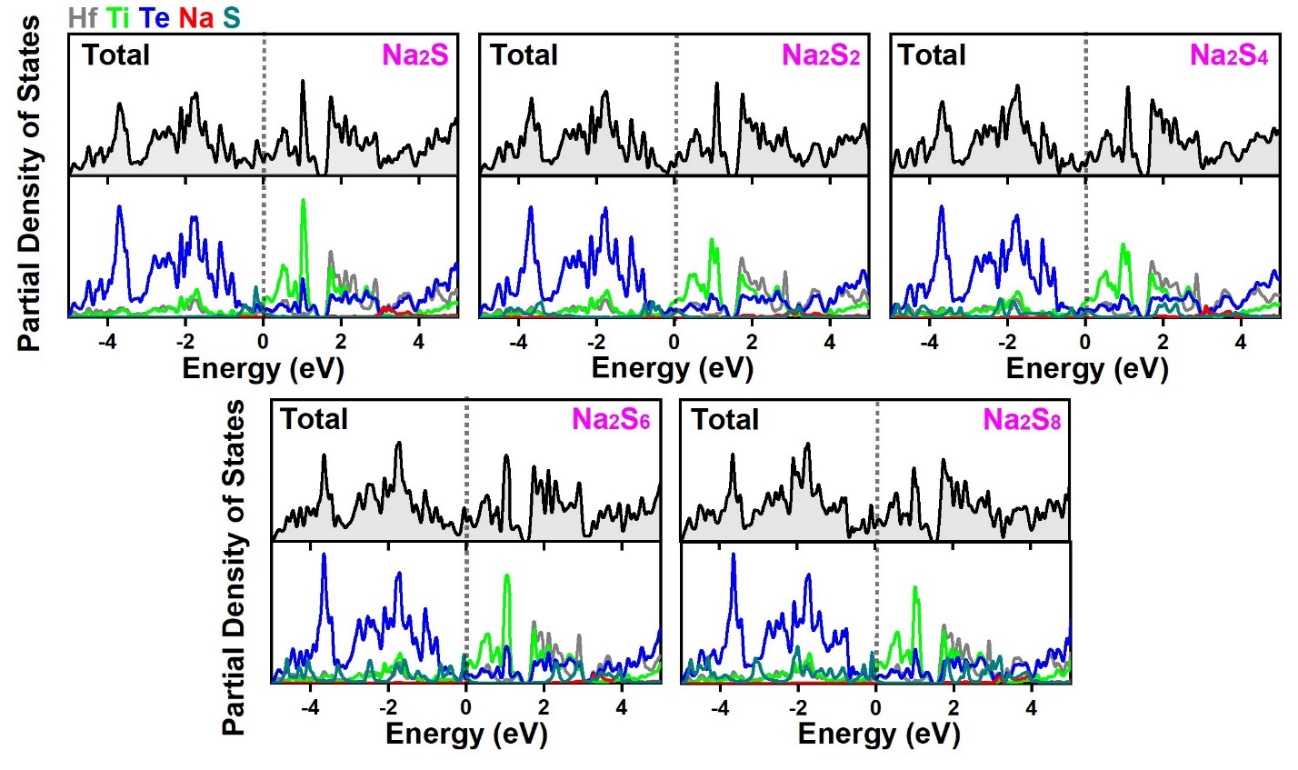


**Figure S15.** Partial densities of states of Na_2_S, Na_2_S_2_, Na_2_S_4_, Na_2_S_6_, and Na_2_S_8_ adsorbed HfZrTe_4_ substrate.


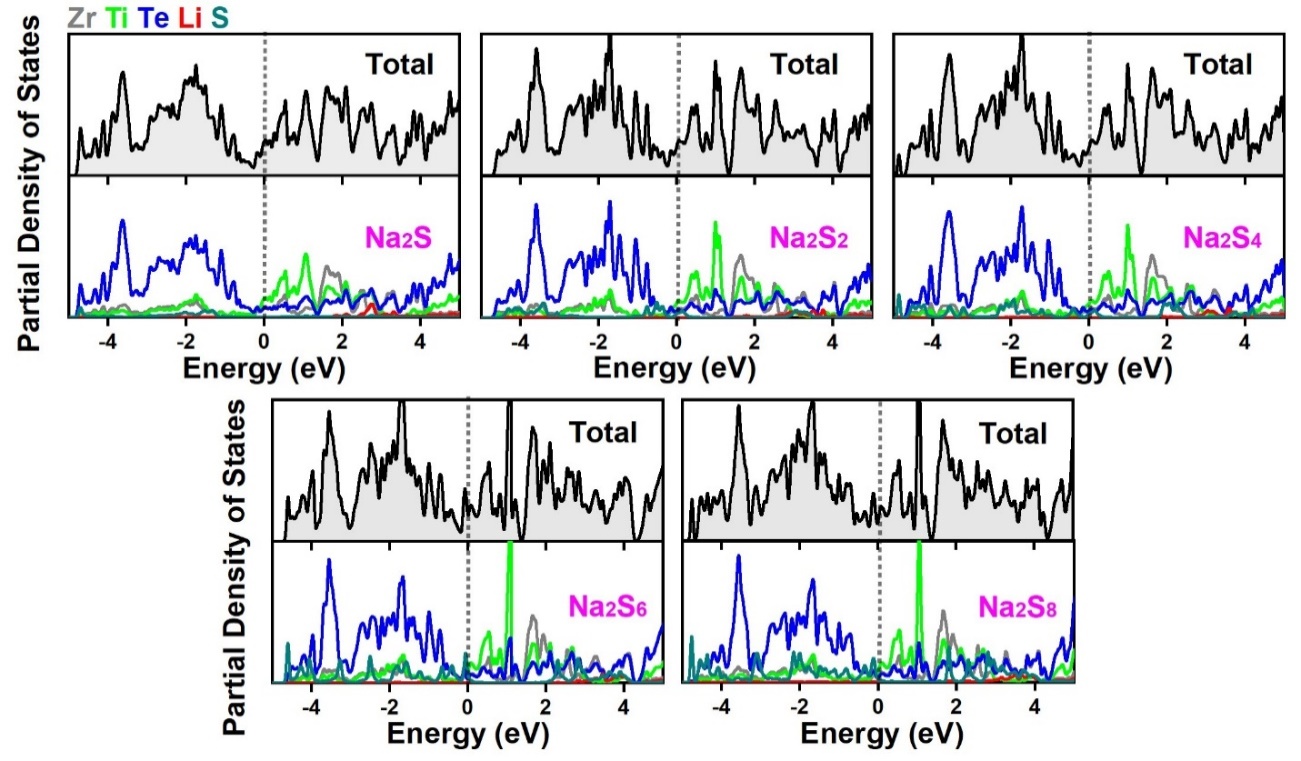


**Figure S16.** Partial densities of states of Na_2_S, Na_2_S_2_, Na_2_S_4_, Na_2_S_6_, and Na_2_S_8_ adsorbed ZrTiTe_4_ substrate.

1. **Li_2_S*_n_* and Na_2_S*_n_* species response to HfZrS_4_, and HfZrSe_4_**


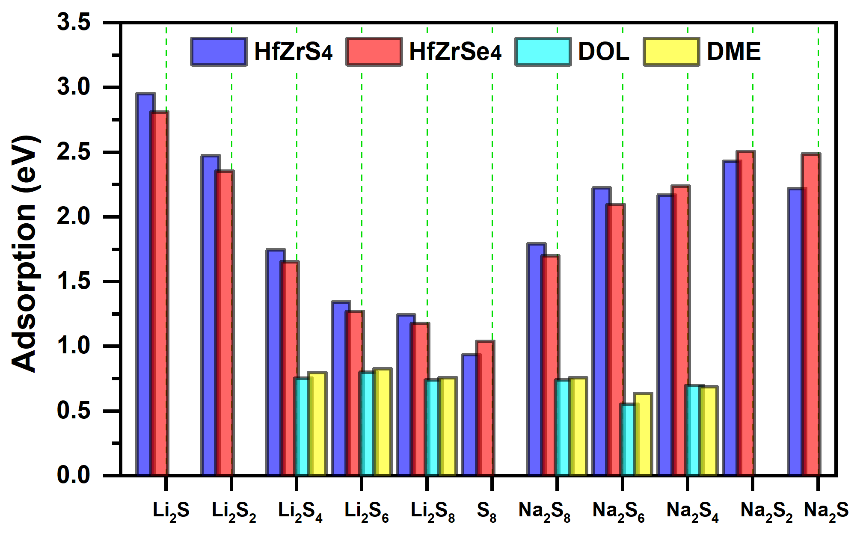


**Figure S17.** Adsorption energies of key lithium and sodium polysulfides species on HfZrS_4_ and HfZrSe_4_ hosts. Bar chart compares binding of S_8_ and Li_2_S*_n_*/Na_2_S*_n_* species (*n* = 1, 2, 4, 6, and 8) on HfZrS_4_ and HfZrSe_4_ monolayers versus the commonly used liquid electrolytes *i.e*., DOL and DME. Higher values adsorption energies for HfZrS_4_/HfZrSe_4_ versus DOL/DME indicate stronger anchoring of lithium and sodium polysulfides on the hosts, suggesting effective shuttle suppression. Vertical dashed lines separate chemical groups.


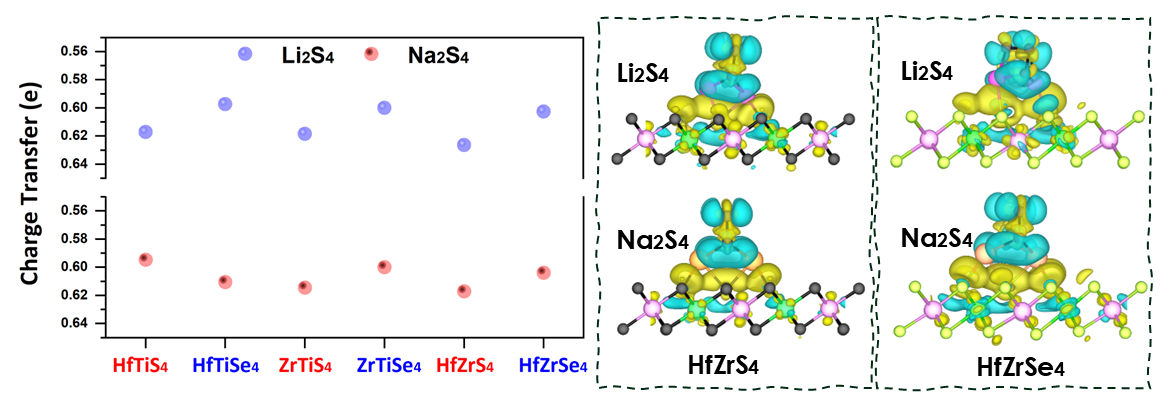


**Figure S18.** Charge transfer (|e|) for Li_2_S_4_ (blue) and Na_2_S_4_ (red) on six hosts (HfTiS_4_, HfTiSe_4_, ZrTiS_4_, ZrTiSe_4_, HfZrS_4_, and HfZrSe_4_), obtained from Bader analysis; larger values indicate more substantial electron donation from the adsorbate to the substrate. Charge–density difference isosurfaces for Li_2_S_4_ and Na_2_S_4_ on HfZrS_4_ and HfZrSe_4_; yellow and cyan denote charge accumulation and depletion, respectively, highlighting interfacial polarization consistent with the charge‐transfer trends.


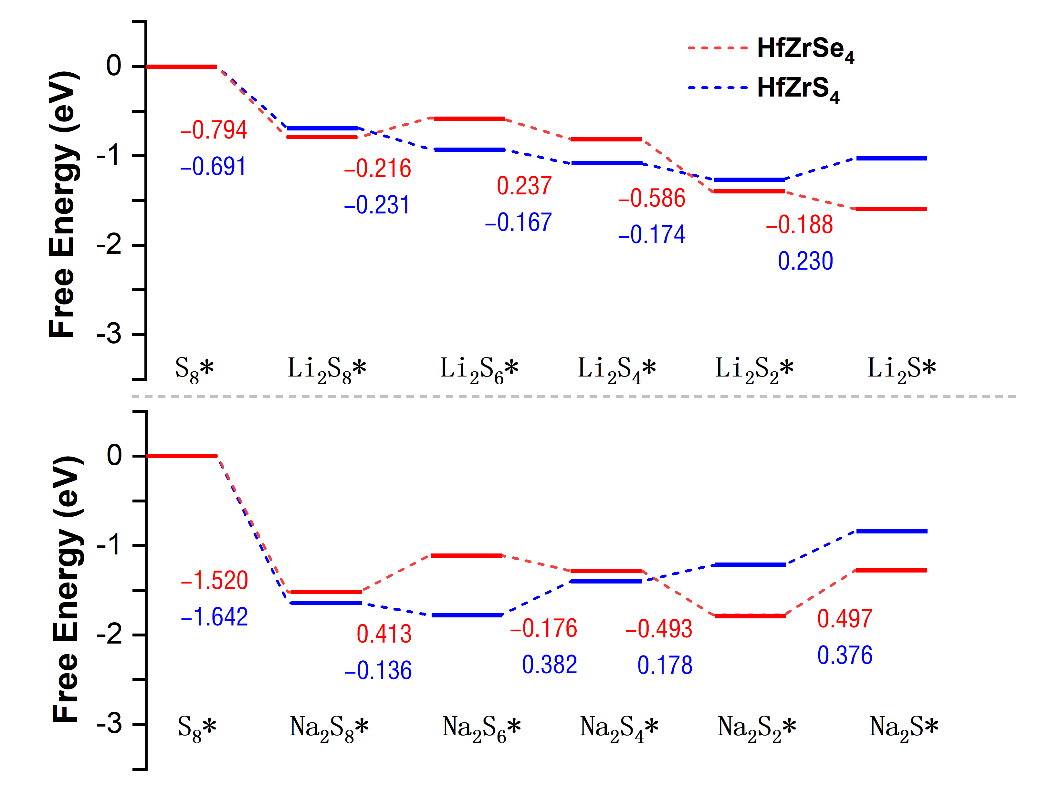


**Figure S19.** Free-energy pathways for the sulfur-reduction reaction S_8_ → Li_2_S on HfZrS_4_ (blue) and HfZrSe_4_ (red). Numbers above edges are step changes; HfZrS_4_ favors early polysulfides, while HfZrSe_4_ stabilizes late-stage species.

1. **Partial densities of states of densely metal-ion adsorbed HfTiTe_4_, HfZrTe_4_, and ZrTiTe_4_.**


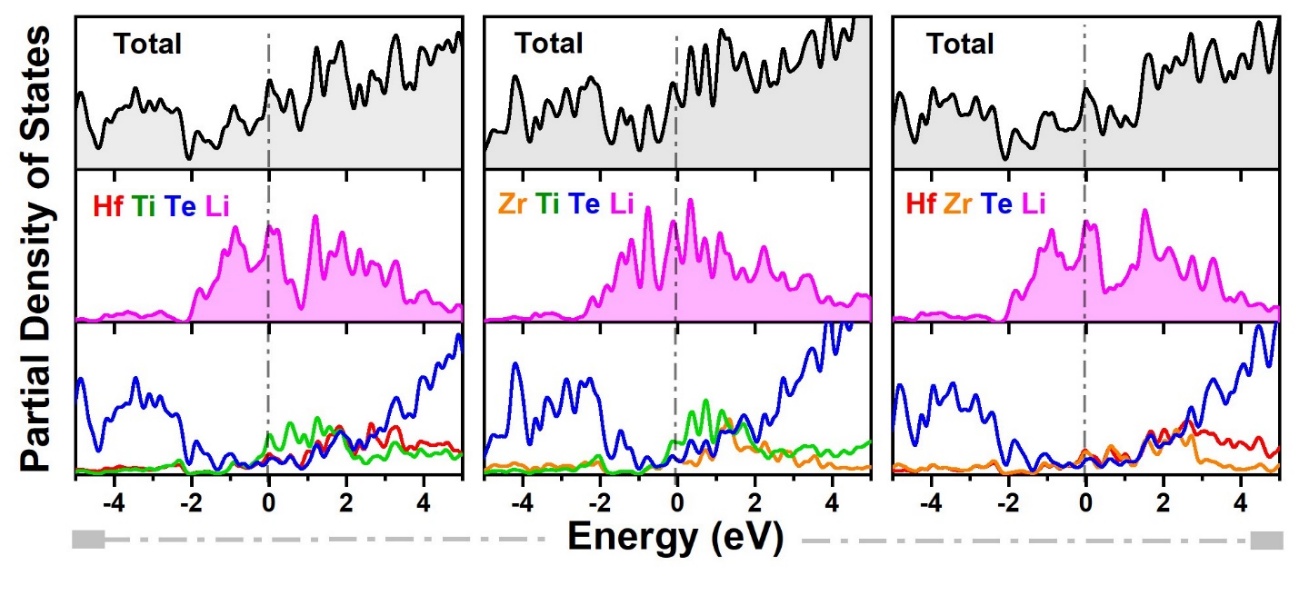


**Figure S20.** Partial densities of states of fully lithium-ion adsorbed **HfTiTe₄, ZrTiTe₄, and HfZrTe₄ monolayers.**


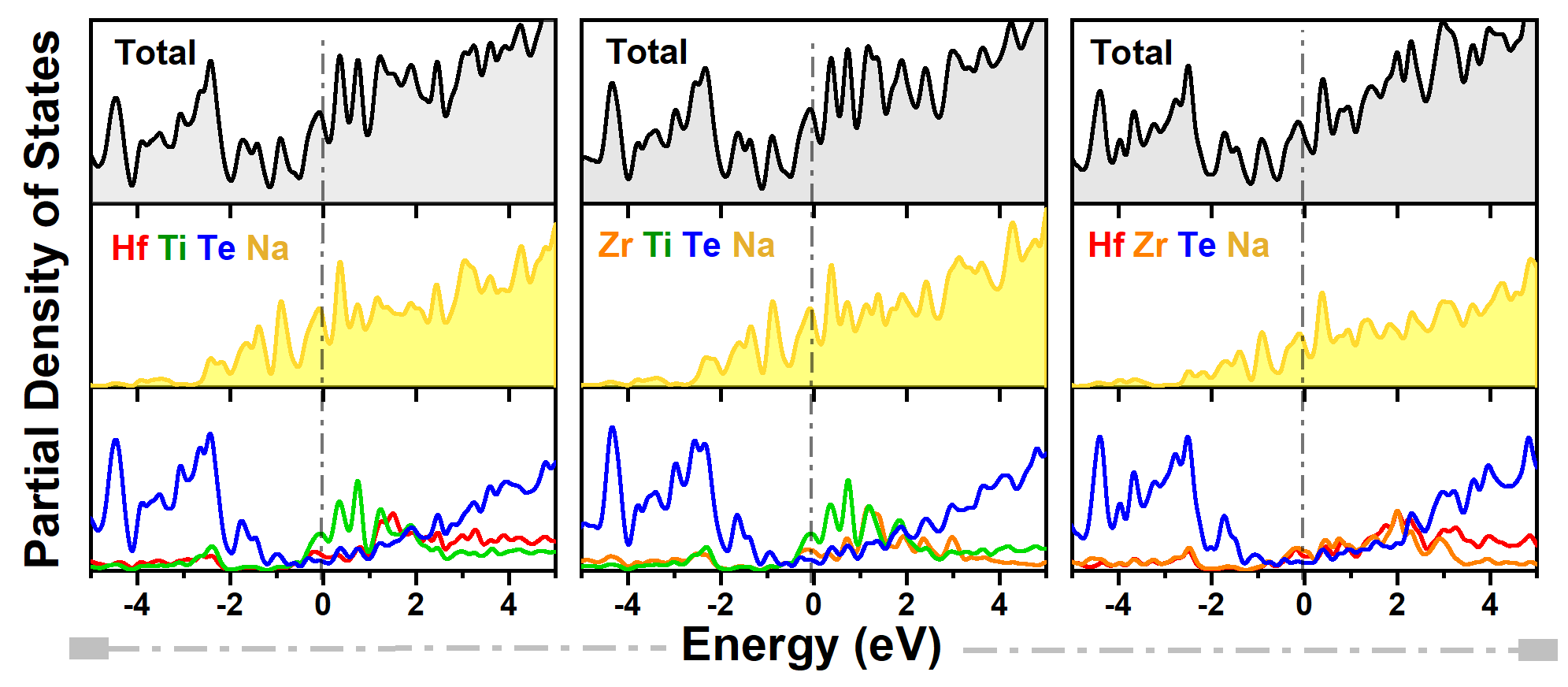


**Figure S21.** Partial densities of states of fully sodium-ion adsorbed **HfTiTe₄, ZrTiTe₄, and HfZrTe₄ monolayers.**

1. **AIMD simulations for Li_2_S and Na_2_S clusters response on monolayer HfZrTe_4_ at 300 K for 20 ps.**


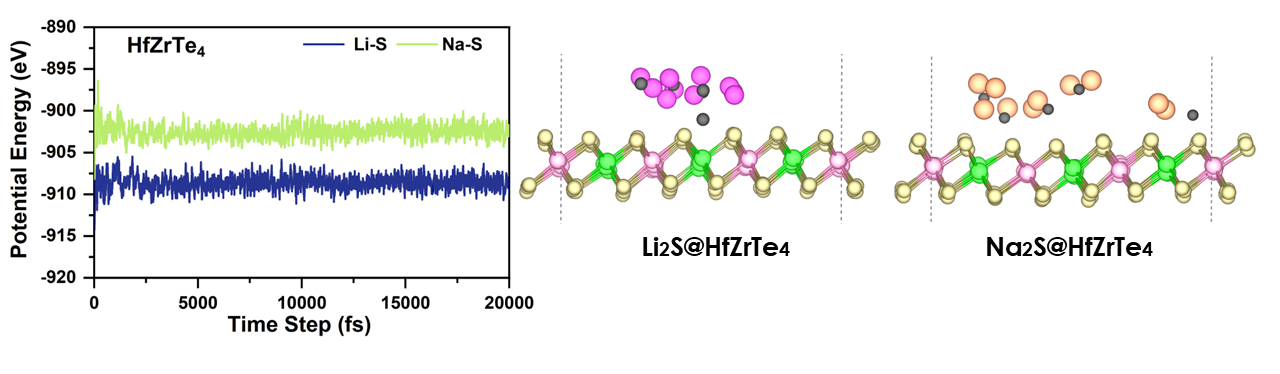


**Figure S22.** The fluctuations of potential energy during the AIMD simulations for 20 psec at 300 K of five Li_2_S and Na_2_S clusters on monolayer HfZrTe_4_ with their corresponding snapshots.
